# The Use of Microtechnology to Quantify the Peak Match Demands of the Football Codes: A Systematic Review

**DOI:** 10.1007/s40279-018-0965-6

**Published:** 2018-08-07

**Authors:** Sarah Whitehead, Kevin Till, Dan Weaving, Ben Jones

**Affiliations:** 1 Institute for Sport, Physical Activity and Leisure, Leeds Beckett University, Leeds, LS6 3QS UK; 2 Leeds Rhinos Rugby League Club, Leeds, UK; 3 Yorkshire Carnegie Rugby Union Club, Leeds, UK; 4 The Rugby Football League, Leeds, UK

## Abstract

**Background:**

Quantifying the peak match demands within the football codes is useful for the appropriate prescription of external training load. Wearable microtechnology devices can be used to identify the peak match demands, although various methodologies exist at present.

**Objectives:**

This systematic review aimed to identify the methodologies and microtechnology-derived variables used to determine the peak match demands, and to summarise current data on the peak match demands in the football codes.

**Methods:**

A systematic search of electronic databases was performed from earliest record to May 2018; keywords relating to microtechnology, peak match demands and football codes were used.

**Results:**

Twenty-seven studies met the eligibility criteria. Six football codes were reported: rugby league (*n* = 7), rugby union (*n* = 5), rugby sevens (*n* = 4), soccer (*n* = 6), Australian Football (*n* = 2) and Gaelic Football (*n* = 3). Three methodologies were identified: moving averages, segmental and ‘ball in play’. The moving averages is the most commonly used (63%) and superior method, identifying higher peak demands than other methods. The most commonly used variables were relative distance covered (63%) and external load in specified speed zones (57%).

**Conclusion:**

This systematic review has identified moving averages to be the most appropriate method for identifying the peak match demands in the football codes. Practitioners and researchers should choose the most relevant duration-specific period and microtechnology-derived variable for their specific needs. The code specific peak match demands revealed can be used for the prescription of conditioning drills and training intensity.

**Electronic supplementary material:**

The online version of this article (10.1007/s40279-018-0965-6) contains supplementary material, which is available to authorized users.

## Key Points

**Table:** 

This review has identified three methods currently used to quantify the peak match demands of the football codes: segmental, moving averages and longest period of ball in play. Practitioners and researchers with the time and skills should use moving averages as the superior method, due to its ability to capture the subtle fluctuations in intensity.
A range of duration-specific periods and microtechnology-derived variables are currently used to identify the peak match demands. These should be selected specific to the needs of the practitioner and/or researcher. Practitioners should consider both short and long durations for the prescription of conditioning drills and monitoring of training intensity during technical-tactical drills.
Given the differences in peak match-demands between codes, prescription of training should be football code and position specific. The highest velocity-based running demands are reported for Gaelic Football, followed by Australian Football; however, the peak acceleration/deceleration demands reported are greatest in rugby league. Positional differences exist across all the football codes, and differences are dependent upon the variables investigated.

## Introduction

Over recent years research into the match demands of the football codes (i.e. soccer, rugby union, rugby sevens, rugby league, Australian Football and Gaelic Football) has increased substantially [1–4]. Methods used to quantify match demands have advanced from video and notational analysis to semi-automated multiple-camera systems (e.g. ProZone and Amisco), and further to microtechnology devices [2, 5, 6]. Microtechnology devices incorporating global positioning system (GPS) receivers and micro-electrical mechanical systems (MEMs) provide researchers and practitioners with valid, reliable and practical methods to quantify the external load players encounter in matches and training [7]. Importantly, this may provide practitioners with information that can optimise the prescription of the external training load, particularly during technical-tactical training [8]. The assessment, and manipulation, of external load is a key process in providing a training stimulus that promotes adaptations whilst minimising negative outcomes (e.g. risk of injury) [9], and consequently increases the likelihood of favourable training outcomes, such as improvements in physical qualities or performance [10].

The integration of GPS and MEMs in microtechnology devices provides practitioners with a plethora of external load variables. The GPS is a navigational system comprising 27 orbiting satellites equipped with atomic clocks, allowing the quantification of movement variables from athletes via the calculation of instantaneous speed [11]. Recent developments in microtechnology devices have also enabled the use of the Global Navigation Satellite System (GNSS) [12]. The GNSS provides geospatial positioning with global coverage, encompassing both the GPS and GLONASS (Global Navigation Satellite System) [12]. Global positioning system-derived variables include basic components of locomotion, including total and relative distance travelled in different speed zones, maximum velocity and accelerations. Micro-electrical mechanical systems include tri-axial accelerometers, gyroscopes and magnetometers, which enable some devices to provide a valid count of collisions [13] and manufacturer specific parameters such as PlayerLoad™ and BodyLoad™ developed from specific algorithms. The advancement of microtechnology devices through sampling rate (1, 5 and 10 Hz), microprocessor and software improvements has provided researchers and practitioners with devices that are deemed both valid and reliable for the measurement of external loads in team sports [14–19]. A review of the validity and reliability of microtechnology has been carried out by Scott et al. [17], indicating 10-Hz devices to be the optimal GPS tracking device, with improvements in accuracy and inter-unit reliability compared to 1- and 5-Hz devices. Despite the good to moderate intra-unit reliability of 10-Hz devices in reporting short distances covered at high velocities, the inter-unit reliability for high-speed and very high-speed running is still limited [20]. Initial research into the GNSS-enabled devices suggests small improvements in interunit reliability when measuring total distance, peak and average speed; however, they are yet to be compared against a criterion measure [12].

Microtechnology devices are now widely used across the football codes by both researchers and practitioners [1, 7, 21] to quantify the volume, intensity, frequency and composition of match activities (e.g. walking, sprinting, accelerating, collisions) undertaken by players (i.e. the external load) [10]. The use of the devices during match play can provide a comprehensive picture of the external load that athletes encounter [22]. For example, half- and whole-match demands [23, 24], positional differences [24], temporal fatigue [25] and match-to-match variation in demands [26] can all be identified.

A commonly used method of analysis for microtechnology data across the football codes is ‘absolute’ match demands, where data are reported as totals or averages for the whole- and or half-match [1]. For example, total distance would be the distance accumulated over the whole-match, and relative distance (distance travelled per minute) would be that total distance divided by the playing time for each player, providing an average for the whole-match. This method of analysis provides some indication of the total external load that players are exposed to during match play and such research has revealed differences in the absolute demands between competitions/levels of play [27–29] and positional groups in several football codes [18, 23, 30]. For example, Brewer et al. [27] revealed professional Australian Football players covered ~ 12 km during match-play, with the average ‘intensity’ (i.e. relative distance covered) being 9% higher at the elite level than the sub-elite level (128 ± 12 vs. 117 ± 15 m·min^−1^). In rugby league, hit up forwards have been found to cover lower total distances (~ 3569 m) during match-play compared to wide-running forwards (~ 5561 m), adjustables (~ 6411 m) and outside backs (~ 6819 m), with outside backs covering more high speed running than all other positions [23]. Such information is important for practice such as assisting with the progression of players from sub-elite to elite competition by preparing them for the likely greater intensity and volume of external loads encountered.

However, the information provided from the absolute demands about match-play can be limited. The intermittent nature of the football codes means averaging across a whole- or half-match provides a blunt measure of physical demands of the sport. For example, in rugby league whole-match intensities of ~ 90 m·min^−1^ have been reported [30], which equate to an average speed of 5.4 km·h^−1^. But it is known that rugby league is intermittent, involving bouts of high speed running and sprinting [23, 31, 32], thus the use of absolute values averaged over a whole match likely underrepresents prolonged (i.e. > 5 min) periods of intense activity which might be important to the outcome of a match [33]. This is also evident in research from soccer using computerised time-motion analysis of match play. Mohr et al. [34] revealed that when the match is split into 5-min periods the average of the distance covered at high-intensity running of these periods is 121 ± 4 m, compared to 219 ± 8 m for the peak period (identified as the 5-min block with the most high-intensity running). Considering this, several researchers have aimed to identify the ‘peak’ demands of the football codes, using microtechnology and different arbitrary temporal durations, from 1 to 10 min [35–37]. By breaking down match play into shorter periods, the most intense periods of play can be identified, assisting practitioners to develop more appropriate drills and training prescription. The importance of investigating and preparing players for the ‘peak periods’ is evident as the most intense periods of play often occur at critical periods of match play. For example, in rugby league Gabbett et al. [33] found the highest number of repeated high-intensity bouts per minute to occur when players were defending their own try line.

To identify the peak match demands using microtechnology, several methodologies have been used, including different temporal durations, analysis techniques and microtechnology derived variables. Considering the importance of identifying and quantifying the peak match demands, researchers and practitioners need to be aware of the different methodologies utilised in research and their transference to practice. Furthermore, through the summary of current research on the peak match demands of football codes, practitioners will have duration-specific target intensities that can be used for the prescription of conditioning drills and monitoring the intensity of coach-led drills to ensure optimal preparation for match play. Therefore, the purpose of this systematic review was to: (1) determine the methodologies utilised to quantify the peak match demands within the football codes; (2) identify the GPS and MEMs variables reported for peak match demands; and (3) summarise the peak-match demands of the football codes.

## Methods

### Design and Search Strategy

A systematic review was performed in accordance with the Preferred Reporting Items of Systematic Reviews and Meta-analyses (PRISMA) statement [38]. A systematic search of electronic databases (Web of Science, SPORTDiscus, CINAHL, MEDLINE and Scopus) was performed from the earliest record to May 2018. All study designs were included. The search strategy combined terms covering the topics of microtechnology devices (GPS OR ‘Global positioning systems’ OR ‘micro-technology’ OR ‘microtechnology’ OR ‘micro-electrical mechanical systems’) AND match demands (‘match performance’ OR ‘match play’ OR ‘match demands’ OR ‘match characteristics’ OR ‘physical demands’ OR ‘movement demands’ OR ‘movement characteristics’ OR ‘activity profiles’ OR ‘peak demands’) AND football codes (‘football’ OR ‘soccer’ OR ‘rugby’ OR ‘rugby union’ OR ‘rugby league’ OR ‘rugby sevens’ OR ‘Australian football’ OR ‘Australian rules football’ OR ‘AFL’ OR ‘Gaelic’ OR ‘Gaelic football’). Reference lists of all selected papers were manually searched for other potentially eligible papers.

### Study Selection

After eliminating duplicates, search results were screened independently by two researchers (SW, BJ) against the eligibility criteria. Disagreements were resolved through discussion, or via a third researcher if required. References that could not be eliminated by the title or abstract were retrieved and evaluated for inclusion via full-text. The title and authors were not masked to the reviewers.

Studies were eligible for inclusion if they investigated the peak movement demands of competitive match play in one of the football codes, defined as either having an aim to identify the ‘peak’, ‘hardest’, ‘highest’ or ‘most intense’ periods, or described as doing so in the methods. Studies were included for all levels of play (elite, sub-elite, amateur or junior) and if at least one microtechnology variable was analysed (e.g. total distance covered, accelerations, collisions). Only peer-reviewed papers were included; abstracts and conference papers were not included. Papers from all languages were included but excluded if translations could not be made. Studies were excluded if they investigated the wrong sport (i.e. not one of the classified football codes: soccer, rugby union, rugby league, rugby sevens, Australian Football or Gaelic Football), used the wrong technology (e.g. video or player tracking technology), did not analyse competitive match play or did not aim to identify the peak demands.

### Data Extraction

Data relating to the participant’s characteristics (i.e. sex, age, height, body mass, level of competition), microtechnology specifications (i.e. brand, model, GPS sampling frequency, accelerometer sampling frequency, software), movement demands (i.e. locomotive variables, collisions) and the football code (i.e. soccer, rugby union, rugby sevens, rugby league, Australian Football, Gaelic Football) played were extracted. The methods of analysis (e.g. segmental, moving averages or ball in play) and temporal durations (e.g. 5 min) used to analyse the ‘peak’ periods were extracted. Where necessary, means and measures of dispersion were extracted from figures in the manuscripts using WebPlotDigitizer v3.12 [39]. Two studies [40, 41] did not report the raw peak demand values therefore the data were not extracted but the studies were included in the review to report the methods utilised. Sparks et al. [42] used the moving averages to determine the peak demands but also used segmental analysis to observe changes in intensity over time, therefore the maximum value observed for segmental analysis was also extracted for comparison between methods. For ease of comparison, metrics were converted to the same units as most other studies, i.e. stature is reported in centimetres (cm), body mass in kilograms (kg), distance covered in metres (m), relative distance in metres per minute (m·min^−1^) and running speed in m·s^−1^.

### Assessment of Methodological Quality

The methodological quality of the included studies was assessed using the modified assessment scale of Downs and Black [43] by two researchers (SW, BJ). Other reviews in this research field [1, 3] used this assessment scale, using only 12 (numbers 1–4, 6, 7, 10–12, 16, 18, 20) of the 27 criteria that logically applied. Due to no interventions being carried out in any of the studies included in the review, question 4 was omitted, leaving 11 criteria to be assessed. Question 10 was modified to include the reporting of effect sizes. No studies were eliminated on the basis of methodological quality. A score of ‘0’ for “absent or insufficient information provided” or ‘1’ “item is explicitly described” was assigned to the 11 criteria.

### Statistical Analysis

A meta-analysis was not performed as study designs were heterogeneous thus not able to be pooled. All data are presented as mean ± standard deviation (SD), mean; ± confidence limits (CL) or mean (CL range).

## Results

### Identification and Selection of Studies

**Fig. 1:**
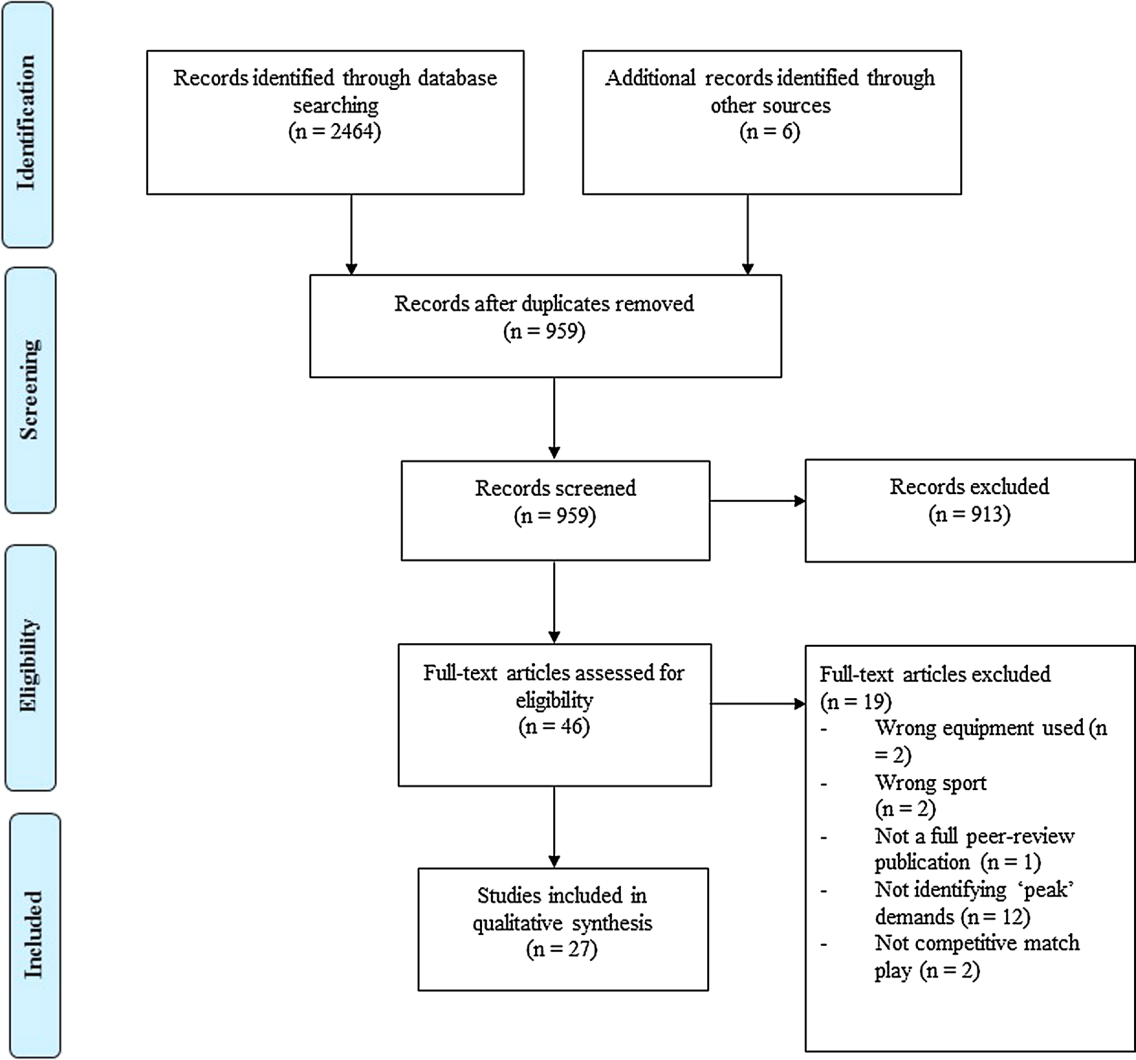
Flow of selection process of eligible studies for qualitative synthesis

Through the original database search 2464 articles were identified, with six others found through other sources. Following the removal of duplicates and screening for eligibility, 27 articles remained for analysis [25, 32, 35, 36, 40–42, 44–63]. Figure 1 provides a schematic representation of the decision process.

### Study Characteristics

Table 1 shows the characteristics of the 27 studies included in the review. Six different football codes were covered: soccer (*n* = 6) [40, 42, 45, 56, 57, 61], Australian Football (*n* = 2) [25, 47], rugby league (*n* = 7) [32, 36, 44, 46, 49, 50, 63], rugby sevens (*n* = 4) [35, 41, 51, 52], rugby union (*n* = 5) [48, 53–55, 58] and Gaelic Football (*n* = 3) [59, 60, 62]. Six studies reported the sex of the participants directly [42, 57, 58, 61–63], 19 studies reported the league/competition that the participants compete in thus the sex could be inferred, and two studies did not report the sex or competition [48, 55]. The playing standard of participants in the studies included international (*n* = 8; 30%), professional club (*n* = 12; 44%) and semi-professional/elite (*n* = 7; 26%). Most studies (*n* = 20; 77%) were carried out with only one team. Information on the microtechnology devices utilised in the study is shown in Table 2.

**Table 1:** Characteristics of the studies included in the review [25, 32, 35, 36, 40–42, 44–63]

Study	Football code	Playing standard	No. of teams	No. of participants	No. of matches	No. of match files	Age (years)	Sex	Body mass (kg)	Stature (cm)
Akenhead et al. (2013) [40]	Soccer	Professional	1	36	18	Not reported	19.3 ± 0.5	Male	77.9 ± 7.4	183 ± 5
Black et al. (2016) [25]	Australian Football	Professional	1	24	13	163	24.1 ± 3.5	Male	88.5 ± 9.5	187 ± 8
Carling et al. (2017) [54]	Rugby union	International	2	63	10	226	19.8 ± 0.5	Male	99.1 ± 9.1	185 ± 7
Couderc et al. (2017) [41]	Rugby sevens	International	1	12	7	Not reported	26.2 ± 3.7	Male	90.6 ± 12.5	184 ± 9
Cunningham et al. (2018) [55]	Rugby union	International	3	119	36	708	Forwards: 24 ± 4 Backs: 23 ± 4	Not reported	Forwards: 111.3 ± 9.3 Backs: 90.0 ± 8.1	Forwards: 189 ± 7 Backs: 181 ± 6
Delaney et al. (2015) [36]	Rugby league	Professional	1	32	20	297	26 ± 4.8	Male	99.1 ± 9.6	184 ± 6
Delaney et al. (2016) [46]	Rugby league	Professional	1	37	43	612	27 ± 5.1	Male	98.5 ± 8.8	184 ± 5
Delaney et al. (2017) [48]	Rugby union	International	2	67	33	570	27.3 ± 3.1	Not reported	104.7 ± 13.9	187 ± 8
Delaney et al. (2017) [47]	Australian Football	Professional	1	40	30	623	24 ± 3	Male	87.9 ± 5.4	191 ± 4
Delaney et al. (2017) [56]	Soccer	Professional	1	24	40	434	24.4 ± 5.4	Male	75.2 ± 5.8	179 ± 6
Furlan et al. (2015) [51]	Rugby sevens	International	1	12	6	61	21.5 ± 2.9	Male	90.1 ± 8.4	185 ± 60
Grantatelli et al. (2014) [52]	Rugby sevens	Professional	1	9	15	Not reported	25.1 ± 3.1	Male	86.0 ± 9.4	181 ± 4
Hulin and Gabbett (2015) [49]	Rugby league	Semi-professional	4	77	20	200	23.9 ± 3.2	Male	Not reported	Not reported
Hulin et al. (2015) [32]	Rugby league	Professional	2	31	25	200	Not reported	Male	Not reported	Not reported
Kempton et al. (2013) [44]	Rugby league	Professional	1	17	45	118	Senior: 22.3 ± 2.5 Junior: 18.0 ± 1.2	Male	Senior: 86.2 ± 5.8 Junior: 82.6 ± 3.6	Senior: 179 ± 5 Junior: 178 ± 6
		(senior and junior)								
Kempton et al. (2015) [50]	Rugby league	Professional	1	18	38	165	24.2 ± 3.6	Male	96.4 ± 7.3	184 ± 6
Malone et al. (2017) [62]	Gaelic Football	Elite amateur	1	32	30	300	24 ± 6	Male	81 ± 7	180 ± 7
Murray and Varley (2015) [35]	Rugby sevens	International	1	17	24	143	Not reported	Male	Not reported	Not reported
Ramos et al. (2017) [57]	Soccer	International	1	12	7	Not reported	18 ± 0.7	Female	62.0 ± 6.2	167 ± 5.8
Read et al. (2018) [58]	Rugby union	Junior elite	7	202	24	472	17.7 ± 0.6	Male	90.8 ± 12.0	183 ± 6
Reardon et al. (2017) [53]	Rugby union	Professional	1	39	17	200	27.2 ± 3.9	Male	99.2 ± 24.4	185 ± 43
Ryan et al. (2018) [59]	Gaelic Football	Elite amateur	1	36	19	154	24 ± 6	Male	81 ± 7	180 ± 7
Ryan et al. (2018) [60]	Gaelic Football	Elite amateur	1	35	19	154	24 ± 6	Male	81 ± 7	180 ± 7
Sparks et al. (2016) [42]	Soccer	Semi-professional	1	10	12	Not reported	22.1 ± 2.5	Male	63.5 ± 9.6	173 ± 8
Trewin et al. (2017) [61]	Soccer	International	1	45	55	606	Not reported	Female	Not reported	Not reported
Varley et al. (2012) [45]	Soccer	Professional	1	19	11	77	Not reported	Male	Not reported	Not reported
Whitehead et al. (2018) [63]	Rugby league	Junior elite and international	1	48	6	102	Club: 15.5 ± 0.7 International: 15.8 ± 0.5	Male	Club: 81.9 ± 12.8 International: 81.1 ± 5.0	Club: 178 ± 6 International: 178 ± 6

Data are expressed as mean ± SD

**Table 2:** Micro-technology hardware and software specification used by studies included in this review

Study	Brand	Model	GPS sampling frequency	Software
Akenhead et al. (2013) [40]	Catapult	MinimaxX	10 Hz	Logan Plus v4.5
Black et al. (2016) [25]	Catapult	MinimaxX S4	10 Hz	Not reported
Carling et al. (2017) [54]	STATSport	Viper 2	10 Hz	Viper Rugby v 2.6.1.173
Couderc et al. (2017) [41]	Digital Simulation	SensorEverywhere	8 Hz	SensorEverywhere
Cunningham et al. (2018) [55]	STATSport	Viper Pod	10 Hz	Not reported
Delaney et al. (2015) [36]	GPSports	SPI HPU	5 Hz (interpolated to 15 Hz)	Team AMS
Delaney et al. (2016) [46]	GPSports	SPI HPU	5 Hz (interpolated to 15 Hz)	Team AMS
Delaney et al. (2017) [48]	GPSports	SPI HPU	5 Hz (interpolated to 15 Hz)	Team AMS v2016.1
Delaney et al. (2017) [47]	Catapult	MinimaxX S5	10 Hz	Openfield v1.12.0
Delaney et al. (2017) [56]	Catapult	OptimEye S5	10 Hz	Openfield v1.11.1
Furlan et al. (2015) [51]	GPSports	SPI HPU	5 Hz (interpolated to 15 Hz)	Labview 2011 (custom written software)
Granatelli et al. (2014) [52]	GPSports	SPI Elite	1 Hz	Team AMS v1.2
Hulin and Gabbett (2015) [49]	Catapult	MinimaxX	10 Hz	Sprint v5.1.0.1
Hulin et al. (2015) [32]	Catapult	MinimaxX S4	10 Hz	Sprint v5.1.0.1
Kempton et al. (2013) [44]	GPSports	SPI Pro	5 Hz	Team AMS v2.1
Kempton et al. (2015) [50]	GPSports	SPI Pro	5 Hz	Team AMS R1 2012.1
Malone et al. (2017) [62]	VXSport	Not reported	4 Hz	View
Murray and Varley (2015) [35]	Catapult	MinimaxX S4	10 Hz	Not reported
Ramos et al. (2017) [57]	Catapult	MinimaxX S5	10 Hz	Not reported
Read et al. (2018) [58]	Catapult	OptimEye S5	10 Hz	Sprint v5.17
Reardon et al. (2017) [53]	Catapult	MinimaxX S5	10 Hz	Sprint v5.1
Ryan et al. (2018) [59]	VXSport	Not reported	4 Hz	View
Ryan et al. (2018) [60]	VXSport	Not reported	4 Hz	View
Sparks et al. (2016) [42]	Catapult	MinimaxX S4	10 Hz	Logan Plus v4.7.1
Trewin et al. (2018) [61]	Catapult	MinimaxX S4	10 Hz	Sprint v5.1
Varley et al. (2012) [45]	GPSports	SPI Pro	5 Hz	Not reported
Whitehead et al. (2018) [63]	Catapult	OptimEye S5	10 Hz	Openfield v1.14

### Methodological Quality

The scores for the assessment of methodological quality are shown in Table 3, ranging from 7 to 9, out of the 11 items assessed.

**Table 3:** Methodological quality assessment (Downs and Black 1998) [43]

Study	Question number											Total score
	1	2	3	6	7	10	11	12	16	18	20	
Akenhead et al. (2013) [40]	1	1	1	1	1	1	0	0	1	1	0	8
Black et al. (2016) [25]	1	1	1	1	1	1	0	0	1	1	1	9
Carling et al. (2017) [54]	1	1	1	1	1	1	0	0	1	1	1	9
Couderc et al. (2017) [41]	1	1	1	1	1	0	0	0	1	1	1	8
Cunningham et al. (2018) [55]	1	1	1	1	1	0	0	0	1	1	1	8
Delaney et al. (2015) [36]	1	1	1	1	1	1	0	0	1	1	1	9
Delaney et al. (2016) [46]	1	1	1	1	1	0	0	0	1	1	1	8
Delaney et al. (2017) [48]	1	1	1	1	1	1	0	0	1	1	1	9
Delaney et al. (2017) [47]	1	1	1	1	1	1	0	0	1	1	1	9
Delaney et al. (2017) [56]	1	1	1	1	1	0	0	0	1	1	1	8
Furlan et al. (2015) [51]	1	1	1	1	1	1	0	0	1	1	0	8
Grantatelli et al. (2014) [52]	1	1	1	1	1	1	0	0	1	1	0	8
Hulin and Gabbett (2015) [49]	1	1	0	1	1	1	0	0	1	1	1	8
Hulin et al. (2015) [32]	1	1	0	1	1	1	0	0	1	1	1	8
Kempton et al. (2013) [44]	1	1	1	1	1	1	0	0	1	1	1	9
Kempton et al. (2015) [50]	1	1	1	1	1	1	0	0	1	1	1	9
Malone et al. (2017) [62]	1	1	1	1	1	1	0	0	1	1	0	8
Murray and Varley (2015) [35]	1	1	0	1	1	1	0	0	1	1	1	8
Ramos et al. (2017) [57]	1	1	1	1	1	1	0	0	1	1	1	9
Read et al. (2018) [58]	1	1	1	1	1	1	0	0	1	1	1	9
Reardon et al. (2017) [53]	1	1	1	1	1	1	0	0	1	1	1	9
Ryan et al. (2018) [59]	1	1	1	1	1	1	0	0	1	1	0	8
Ryan et al. (2018) [60]	1	1	1	1	1	1	0	0	1	1	0	8
Sparks et al. (2016) [42]	1	1	1	1	1	1	0	0	1	1	1	9
Trewin et al. (2017) [61]	1	1	0	1	1	1	0	0	1	1	1	8
Varley et al. (2012) [45]	1	1	0	1	1	1	0	0	1	1	0	7
Whitehead et al. (2018) [63]	1	1	1	1	1	1	0	0	1	1	1	9

1 = yes, 0 = no or unable to determine (where applicable)

### Methodology for Quantifying the Peak Match Demands

Table 4 shows the different methodologies used by the studies included in the review. Three different methods of analysis were used: segmental analysis, moving averages and the period of longest ball in play, and several different temporal durations. Two studies directly compared two methods (segmental vs. moving average) [45, 55].

**Table 4:** Methods and variables used to quantify peak demands in the football codes [25, 32, 35, 36, 40–42, 44–62]

Study	Football code	Method	Duration (min)	Variables
Akenhead et al. (2013) [40]	Soccer	Segmental	5	Acceleration and deceleration
Black et al. (2016) [25]	Australian Football	Moving average	3	RD, RD in speed zone(s)
Carling et al. (2017) [54]	Rugby union	Moving average	5	HML^a^ distance
Couderc et al. (2017) [41]	Rugby sevens	Moving average	1	TD and RD in speed zone(s)
Cunningham et al. (2018) [55]	Rugby union	Segmental and moving average	1–5	RD, RD in speed zone(s)
Delaney et al. (2015) [36]	Rugby league	Moving average	1–10	RD
Delaney et al. (2016) [46]	Rugby league	Moving average	1–10	Acceleration and deceleration, Met power, RD
Delaney et al. (2017) [48]	Rugby union	Moving average	1–10	Acceleration and deceleration, Met power, RD
Delaney et al. (2017) [47]	Australian Football	Moving average	1–10	Acceleration and deceleration, Met power, RD, RD in speed zone(s)
Delaney et al. (2017) [56]	Soccer	Moving average	1–10	Acceleration and deceleration, Met power, RD, RD in speed zone(s)
Furlan et al. (2015) [51]	Rugby sevens	Moving average	2	Met power, RD
Grantatelli et al. (2014) [52]	Rugby sevens	Segmental	1	RD, percentage distance covered in speed zone(s)
Hulin and Gabbett (2015) [49]	Rugby league	Segmental	5	Collisions^b^, RD, RD in speed zone(s), RHIE^c^
Hulin et al. (2015) [32]	Rugby league	Segmental	5	Collisions^b^, RD, RD in speed zone(s), RHIE^c^
Kempton et al. (2013) [44]	Rugby league	Segmental	5	TD
Kempton et al. (2015) [50]	Rugby league	Moving average	5	Met power, TD, TD in speed zone(s)
Malone et al. (2017) [62]	Gaelic Football	Moving average	1–10	RD, RD in speed zone(s)
Murray and Varley (2015) [35]	Rugby sevens	Moving average	1	Acceleration, TD, TD in speed zone(s)
Ramos et al. (2017) [57]	Soccer	Segmental	5	TD, TD in speed zone(s), Acceleration and deceleration, PlayerLoad^d^
Read et al. (2018) [58]	Rugby union	Moving average	15 and 30 s 1, 2, 2.5, 3, 4, 5 and 10 min	RD
Reardon et al. (2017) [53]	Rugby union	Longest ball in play period	N/A	RD, RD in speed zone(s), TD, *V*_max_, Sprint efforts
Ryan et al. (2018) [59]	Gaelic Football	Segmental	5	Acceleration
Ryan et al. (2018) [60]	Gaelic Football	Segmental	5	Acceleration
Sparks et al. (2016) [42]	Soccer	Segmental and moving average	5	RD in speed zone(s)
Trewin et al. (2017) [61]	Soccer	Moving average	5	TD, RD, RD in speed zone(s), Acceleration, PlayerLoad^d^
Varley et al. (2012) [45]	Soccer	Segmental and moving average	5	TD in speed zone(s)
Whitehead et al. (2018) [63]	Rugby league	Moving average	10 and 30 s 1–10 min	RD

*TD* total distance, *RD* relative distance, *Met Power* metabolic power, *V*_*max*_ maximum velocity

^a^*HML* high metabolic load (high intensity running distance + distance covered while accelerating above 2 m·s^−1^ [53])

^b^*Collisions* any tackles or hit-ups, and decoy runs or support runs where contact is made with a player in the defensive line [67]

^c^*RHIE* repeated high-intensity effort bouts (three or more high-velocity, high-acceleration, or contact efforts with less than 21-s recovery between efforts [23, 32])

^d^*PlayerLoad*™ the accumulation of data from all axes (anteroposterior, mediolateral, and craniocaudal) [68]

#### Segmental

Eleven studies [32, 40, 42, 44, 45, 49, 52, 55, 57, 59, 60] used segmental analysis of pre-determined time periods. This method involved authors specifying the time-period of interest, then splitting the match accordingly following the zero-minute mark. For example, for 5-min blocks a match would be split from 0–5, 5–10, 10–15 min, etc. To determine the peak demands of the match the authors then selected the period with the highest demands of their specified variable(s) of interest.

#### Moving Averages

Varley et al. [45] were the first investigators to use the moving averages method; they directly compared it to the segmental methodology. Subsequently, 16 other studies have used this method [25, 35, 36, 41, 42, 46–48, 50, 51, 54–56, 58, 62, 63]. The moving averages method requires the analysis of the raw instantaneous data, which are sampled at a given rate dependent upon the GPS device used (i.e. a 10-Hz GPS device takes ten instantaneous speed samples per second). To determine the ‘peak’ demands using this method a moving average of a specified duration is taken from the raw data. For example, for 1-min periods a moving average of 600 data points (60 s with ten samples per second) would be calculated from the start to the end of the match, i.e. 0–600, 1–601, 2–602, 3–603, etc., for the duration of the file, and the peak 1-min identified from this.

#### Ball in Play

Reardon et al. [53] is the only study to use the ‘ball in play’ method. They defined the peak demands by identifying the longest period of time when the ball is in play, then extracted the physical demands within this period.

#### Duration

A range of durations were used to quantify peak demands in the studies included in the review as shown in Table 4; the most commonly used duration was 5 min, used by 78% (*n* = 21) of studies. Nine studies used multiple duration-specific periods [36, 46–48, 55, 56, 58, 62, 63], and six of these compared the derived peak demands between each duration [36, 46–48, 58, 62].

### Variables Used for Analysing the Peak Match Demands

Table 4 shows the variables used by the studies included in the review. Two or more variables were used by 67% (*n* = 18) of studies. All studies used at least one locomotive variable (i.e. walking, running, sprinting).

#### Total Distance Covered

Total distance is the distance accumulated by a player over the specified time-period and was used by six of the studies identified in this review [35, 44, 50, 53, 57, 61]. Three different durations were used to determine the ‘peak’ total distance covered: 1 min [35], 5 min [44, 50, 57] and the ‘longest period of ball in play’ [53]. Table 5 shows the distances covered in the specified durations and different methods of analysis. Although only five studies reported total distance directly, this could be extrapolated when relative distance was reported for specified time periods.

**Table 5:** Peak total distance covered for different methods of analysis in the synthesised studies [35, 44, 50, 53, 57]

Study	Sport	Level	Sex	Variable	Method of analysis	Total distance (m)		
						1 min	5 min	N/A
Kempton et al. (2013) [44]	Rugby league	Professional	Male	Senior	Segmental		602; ± 52	
				Junior			592; ± 61	
Kempton et al. (2015) [50]	Rugby league	Professional	Male		Moving averages		540 (529–550)	
Ramos et al. (2017) [57]	Soccer	International	Female	Central defenders	Segmental		601 ± 56	
				Fullbacks			653 ± 41	
				Midfielders			594 ± 51	
				Forwards			623 ± 58	
Murray and Varley (2015) [35]	Rugby sevens	International	Male		Moving averages	183 ± 30		
Reardon et al. (2017) [53]	Rugby union	Professional	Male	Tight five forwards	Ball in play			109 (104–114)
				Back row forwards				111 (105–117)
				Inside backs				123 (117–129)
				Outside backs				124 (117–131)

Data are expressed as mean ± SD, mean; ± 95% CI or mean (95% CI range)

#### Relative Distance Covered

**Fig. 2:**
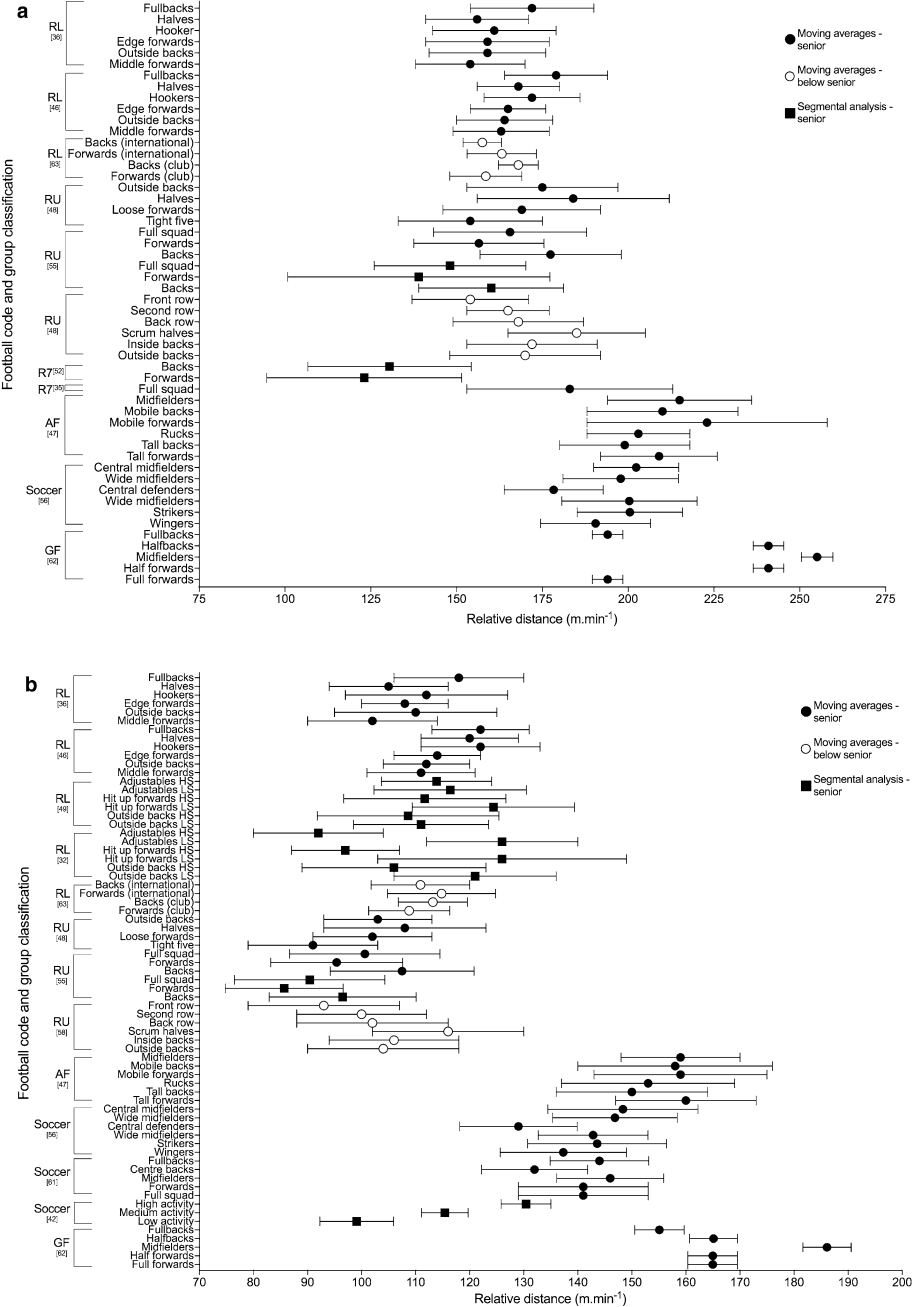

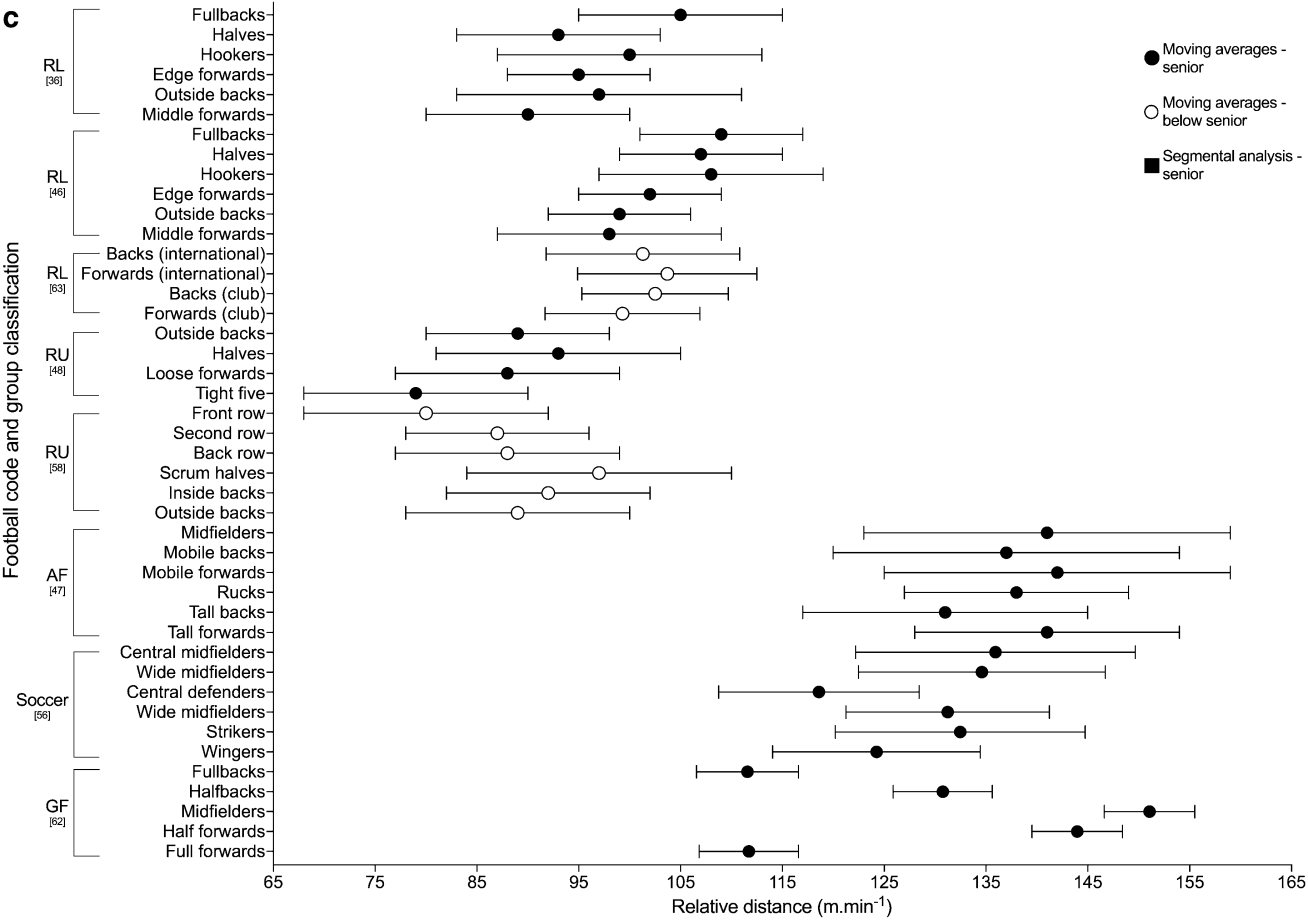
Duration-specific peak relative distance (m·min^−1^) in the football codes. **a** 1 min, **b** 5 min, **c** 10 min. Data are expressed as mean ± SD. *RL* rugby league, *RU* rugby union, *AF* Australian Football, *R7* rugby sevens, *GF* Gaelic Football, *HS* high success (teams that won 71–75% of matches played), *LS* low success (teams that won 32–58% of matches played) [32, 49]

Relative distance (m·min^−1^) is a function of the distance covered relative to the time it is covered in, providing a proxy indication of the ‘intensity’ [1]. It was used by 63% (*n* = 17) of the studies included in the review, with 13 out of the 17 studies using the moving average method. The peak 1-, 5- and 10-min relative distances across the football codes are shown in Fig. 2. All other duration-specific periods are shown in Electronic Supplementary Material Fig. S1. Reardon et al. [53] analysed relative distance within the ‘longest period of ball in play’ in rugby union.

#### External Load Completed Within Speed Zones

Fifty-seven percent (*n* = 16) of studies used variables based on speed zones. Speed zones are categorised by specific thresholds identified by the author. Within the studies included in the review 12 different thresholds were used to categorise ‘high speed’ or ‘high intensity’ running, and four to categorise ‘sprint speed’ or ‘very high intensity’ running. Relative distance covered in specified speed zones was used by 11 studies [25, 32, 41, 42, 47, 49, 55, 56, 61, 62] and total distance covered by four [35, 45, 50, 53]. The relative and total distances covered in the specified speed zones for specified durations are shown in Tables 6 and 7. One study used percentage of distance covered at ‘high speed’ per minute of match play [52], and one used the number of efforts in the specified ‘sprint’ threshold [53].

**Table 6:** Relative and total distance in specified speed zones using the moving averages method of analysis [25, 35, 42, 45, 47, 50, 55, 56, 61, 62]

Study	Sport	Level	Variable	High speed^a^										
				Relative distance (m·min^−1^)										Total distance (m)
				1 min	2 min	3 min	4 min	5 min	6 min	7 min	8 min	9 min	10 min	5 min
Black et al. (2016) [25]	Australian Football	Professional	More experienced^b^			48 ± 17								
			Less experienced^b^			44 ± 16								
Cunningham et al. (2018) [55]	Rugby union	International	Full squad	54 ± 25		25 ± 16		18 ± 12						
			Forwards	43 ± 21		19 ± 14		13 ± 10						
			Backs	70 ± 22		33 ± 14		24 ± 11						
Delaney et al. (2017) [47]	Australian Football	Professional	Midfielders	95 ± 30	58 ± 19	45 ± 15	39 ± 14	34 ± 12	31 ± 11	29 ± 10	27 ± 10	25 ± 9	24 ±9	
			Mobile backs	94 ± 29	58 ± 17	46 ± 14	38 ± 11	34 ± 10	31 ± 9	29 ± 8	27 ± 8	26 ± 7	24 ± 7	
			Mobile forwards	110 ± 45	71 ± 38	55 ± 25	47 ± 20	41 ± 16	37 ± 15	35 ± 13	33 ± 13	31 ± 11	30 ± 11	
			Rucks	70 ± 20	40 ± 11	29 ± 10	23 ± 8	20 ± 7	17 ± 5	15 ± 5	14 ± 4	14 ± 4	13 ± 4	
			Tall backs	76 ± 23	47 ± 15	35 ± 10	28 ± 9	25 ± 8	23 ± 7	21 ± 6	19 ± 6	18 ± 6	17 ± 6	
			Tall Forwards	95 ± 24	58 ± 14	45 ± 11	37 ± 9	33 ± 8	30 ± 7	27 ± 6	25 ± 6	24 ± 5	23 ± 5	
Delaney et al. (2017) [56]	Soccer	Professional	Central midfielders	53 ± 14	32 ± 8	24 ± 6	20 ± 7	19 ± 7	17 ± 6	16 ± 5	14 ± 5	14 ± 5	13 ± 4	
			Wide midfielders	51 ± 18	30 ± 10	23 ± 8	27 ± 7	17 ± 6	15 ± 6	20 ± 5	13 ± 5	12 ± 4	12 ± 4	
			Central defenders	48 ± 15	28 ± 8	22 ± 6	18 ± 5	16 ± 5	14 ± 4	13 ± 4	12 ± 3	11 ± 3	11 ± 3	
			Wide defenders	67 ± 17	40 ± 11	31 ± 8	27 ± 7	17 ± 6	21 ± 6	20 ± 5	19 ± 5	18 ± 4	17 ± 4	
			Strikers	66 ± 6	40 ± 11	31 ± 8	26 ± 7	24 ± 6	22 ± 6	20 ± 5	19 ± 5	18 ± 5	18 ± 5	
			Wingers	59 ± 17	37 ± 11	29 ± 9	25 ± 7	23 ± 7	20 ± 6	19 ± 5	18 ± 5	17 ± 5	17 ± 4	
Kempton et al. (2015) [50]	Rugby league	Professional												160 (155–164)
Malone et al. (2017) [62]	Gaelic Football	Elite	Fullbacks	37 ± 3	35 ± 2	33 ± 5	29 ± 2	28 ± 3	25 ± 3	20 ± 1	22 ± 3	21 ± 2	20 ± 1	
			Halfbacks	47 ± 3	44 ± 4	40 ± 2	38 ± 3	32 ± 4	30 ± 3	28 ± 3	29 ± 3	28 ± 3	28 ± 3	
			Midfielders	50 ± 4	48 ± 5	48 ± 4	45 ± 3	44 ± 3	40 ± 3	33 ± 3	35 ± 3	33 ± 3	33 ± 3	
			Half forwards	46 ± 4	44 ± 3	40 ± 3	37 ± 2	32 ± 3	31 ± 2	28 ± 2	29 ± 3	28 ± 2	28 ± 2	
			Full forwards	37 ± 4	29 ± 3	28 ± 3	25 ± 3	23 ± 2	22 ± 2	21 ± 2	22 ± 3	21 ± 2	21 ± 2	
Murray and Varley (2015) [35]	Rugby sevens	International		86 ± 30										
Sparks et al. (2016) [42]	Soccer	Semi-professional	Low activity^c^	38 ± 4										
			Medium activity^c^	49 ± 4										
			High activity^c^	59 ± 5										
Trewin et al. (2017) [61]	Soccer	International (female)	Full squad					25 ± 8						
			Fullbacks					31 ± 8						
			Central defenders					20 ± 9						
			Midfielders					25 ± 7						
			Forwards					25 ± 6						
Varley et al. (2012) [45]	Soccer	Professional												177 ± 91

Data expressed as mean ± SD or mean (95% CI range)

^a^High-speed running thresholds: Black et al. [25] > 4.15 m·s^−1^, Cunningham et al. [55] > 5.0 m·s^−1^, Delaney et al. [47, 56] > 5.5 m·s^−1^, Kempton et al. [50] > 4.0 m·s^−1^, Malone et al. [62] > 4.7 m·s^−1^, Murray and Varley [35], and Varley et al. [45] > 4.17 m·s^−1^, Sparks et al. [42] > 3.7 m·s^−1^, Trewin et al. [61] > 4.58 m·s^−1^

^b^More experienced players: ≥ 5 years at the elite level, less experienced players: ≤ 4 years at the elite level [25]

^c^Low-, medium- and high-activity groups were classified based on distance covered in the first half (low: ≤ 30th percentile, medium: 35–65th percentile and high: ≥ 75th percentile) [42]

**Table 7:** Relative and total distance covered in specified speed zones using the segmental and ball in play methods of analysis [32, 42, 45, 49, 53, 55, 57]

Study	Sport	Level	Variable	Relative distance (m·min^−1^)			Total distance (m)		
				5 min		Ball in play	5 min		
				> 3.7 m·s^−1^	> 5.0 m·s^−1^	> 60% individuals *V*_max_	> 4.3 m·s^−1^	> 4.17 m·s^−1^	> 5.6 m·s^−1^
Cunningham et al. (2018) [55]	Rugby union	International	Full squad		15 ± 9				
			Forwards		11 ± 7				
			Backs		10 ± 9				
Hulin and Gabbett (2015) [49]	Rugby league	Semi-professional	Adjustables high success^a^		14 ± 4				
			Adjustables low success^a^		14 ± 5				
			Hit ups forwards high success^a^		10 ± 4				
			Hit ups forwards low success^a^		13 ± 6				
			Outside backs high success^a^		15 ± 5				
			Outside backs low success^a^		14 ± 4				
Hulin et al. (2015) [32]	Rugby league	Professional	Adjustables high success^a^		8 ± 5				
			Adjustables low success^a^		17 ± 7				
			Hit ups forwards high success^a^		9 ± 4				
			Hit ups forwards low success^a^		17 ± 7				
			Outside backs high success^a^		17 ± 3				
			Outside backs low success^a^		22 ± 2				
Sparks et al. (2016) [42]	Soccer	Semi-professional	Low activity^b^	19.9 ± 3.5					
			Medium activity^b^	28.2 ± 12.9					
			High activity^b^	36.3 ± 3.5					
Ramos et al. (2017) [57]	Soccer	International (U20 women)	Central defenders				68.9 ± 15.5		37.1 ± 15.3
			Fullbacks				100 ± 15.7		57.4 ± 16.9
			Midfielders				71.3 ± 17.1		36.4 ± 13.6
			Forwards				91.5 ± 27.8		60.7 ± 14.6
Reardon et al. (2017) [53]	Rugby union	Professional	Tight five forwards			4.9 (3.0–6.9)			
			Back row forwards			6.0 (3.8–8.3)			
			Inside backs			8.1 (6.0–10.2)			
			Outside backs			14.1 (11.6–16.7)			
Varley et al. (2012) [45]	Soccer	Professional						142 ± 24	

Data are expressed as mean ± SD or mean (95% CI)

*V*_*max*_ maximum velocity

^a^*High success* teams that won 71–75% of matches played. *Low success* teams that won 32–58% of matches played [32, 49]

^b^Low-, medium- and high-activity groups were classified based on distance covered in the first half (low: ≤ 30th percentile, medium: 35–65th percentile and high: ≥ 75th percentile) [42]

#### Accelerations/Decelerations

**Fig. 3:**
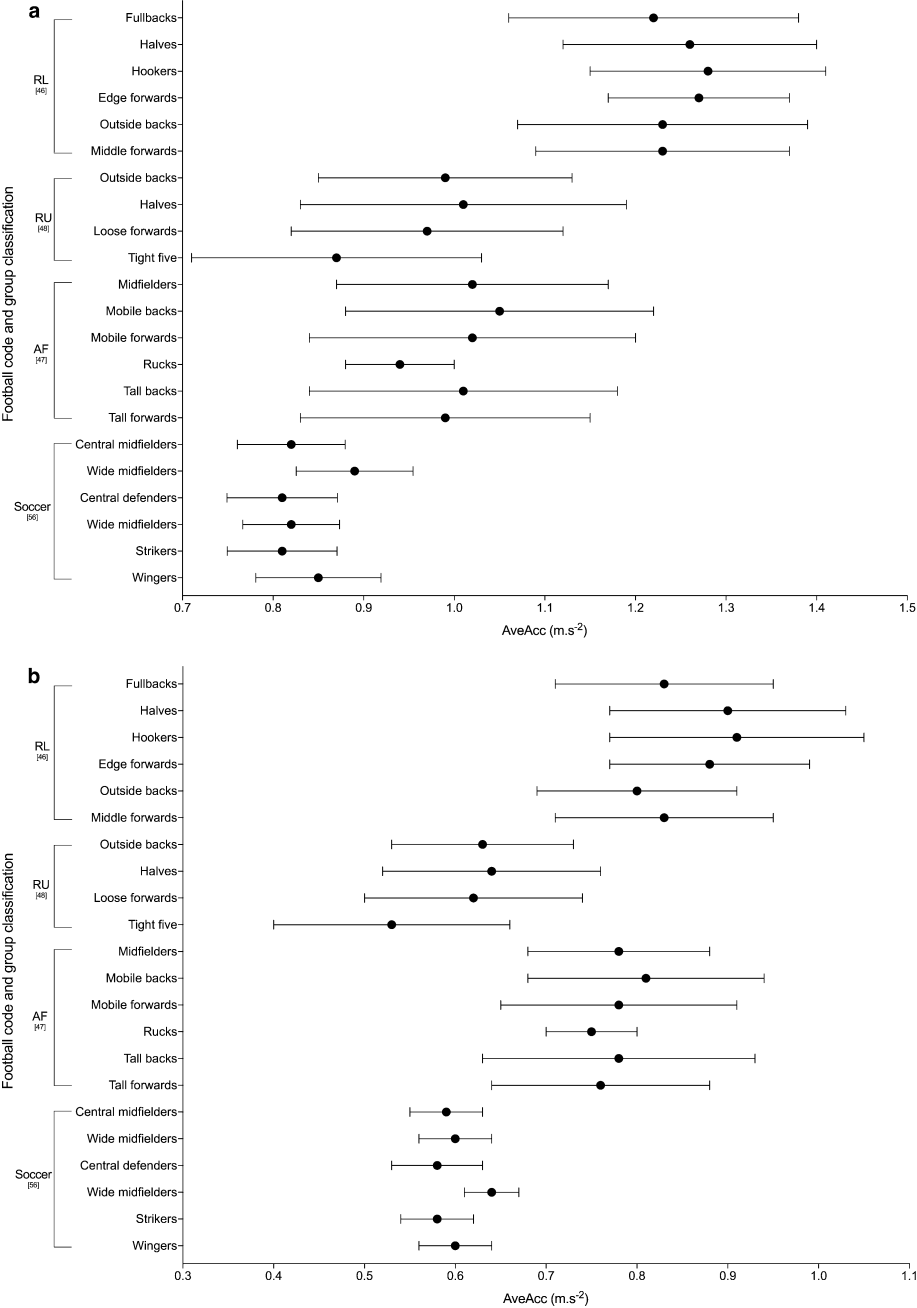

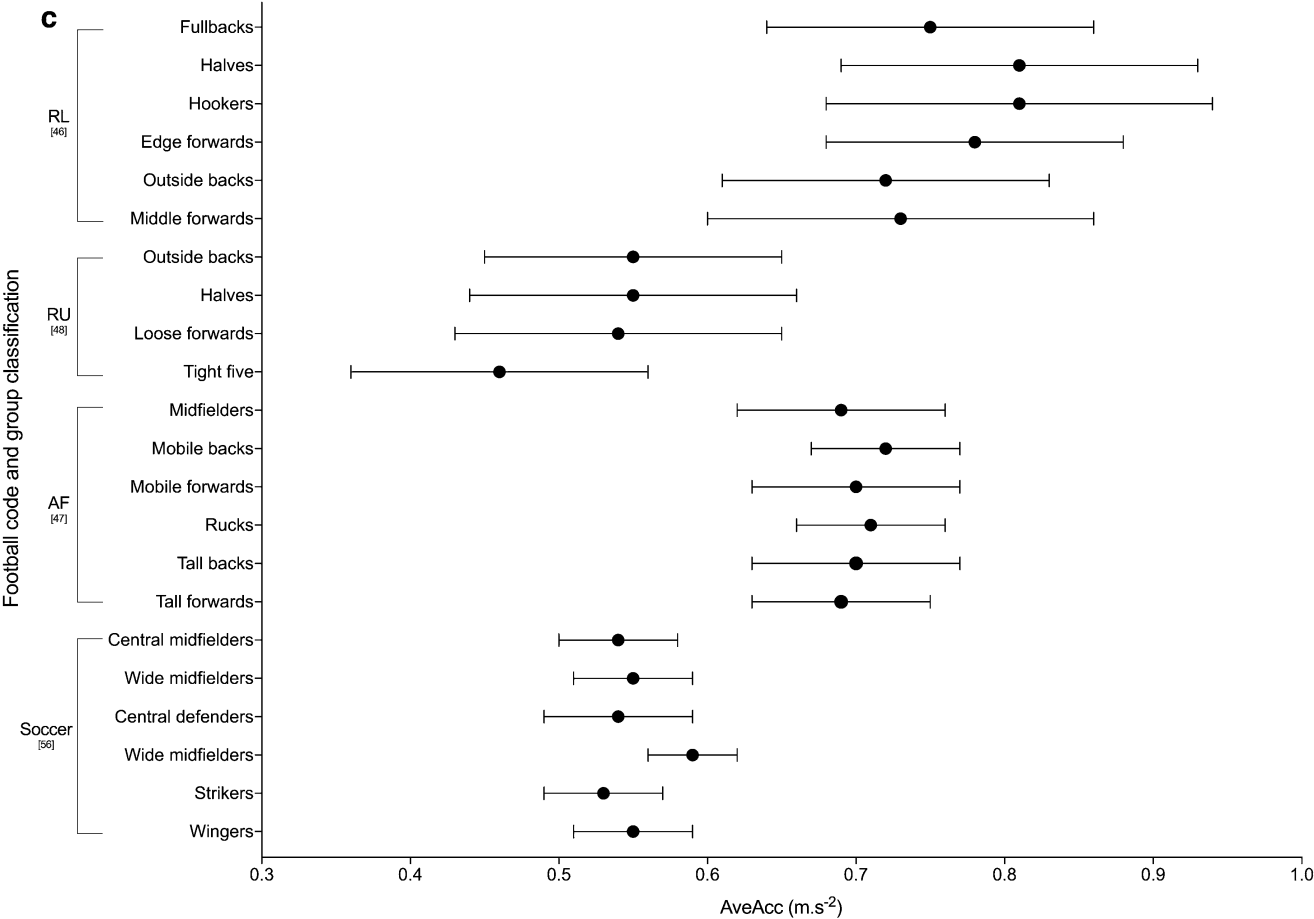
Duration-specific peak average absolute acceleration/deceleration (AveAcc; m·s^−2^) in the football codes. **a** 1 min, **b** 5 min, **c** 10 min. Data are expressed as mean ± SD. *RL* rugby league, *RU* rugby union, *AF* Australian Football

Ten studies used an acceleration and/or deceleration metric to quantify the peak match demands, with three different variables used [35, 40, 46–48, 56, 57, 59–61]. Three studies used distance covered at acceleration to describe the peak 5-min demands [40, 59, 60]. Akenhead et al. [40] used distance covered at high (> 3 m·s^−2^) acceleration and high (< − 3 m·s^−2^) deceleration to describe the peak 5-min demands; however, they only reported percentage change from the mean for the peak demands for these values thus values are not reported. The two studies by Ryan et al. [59, 60] in Gaelic Football both reported acceleration distance only, and at a lower threshold of > 2 km·h^−2^ (0.55 m·s^−2^). Four studies used absolute acceleration/deceleration (AveAcc) as one combined metric [46–48, 56], calculated using the instantaneous acceleration and deceleration of the player (calculated as the rate of change in speed), then taken as an absolute value (i.e. all values being positive) [46]. The studies that used this metric all utilised the moving averages approach to identify duration-specific peak AveAcc demands. The peak 1-, 5- and 10-min AveAc demands are shown in Fig. 3. All other duration-specific periods are shown in Electronic Supplementary Material Fig. S2. The final acceleration variable used was the count of high acceleration occurrences, with each of the three studies using different acceleration speed and minimum duration thresholds [35, 57, 61].

#### Metabolic Power

**Fig. 4:**
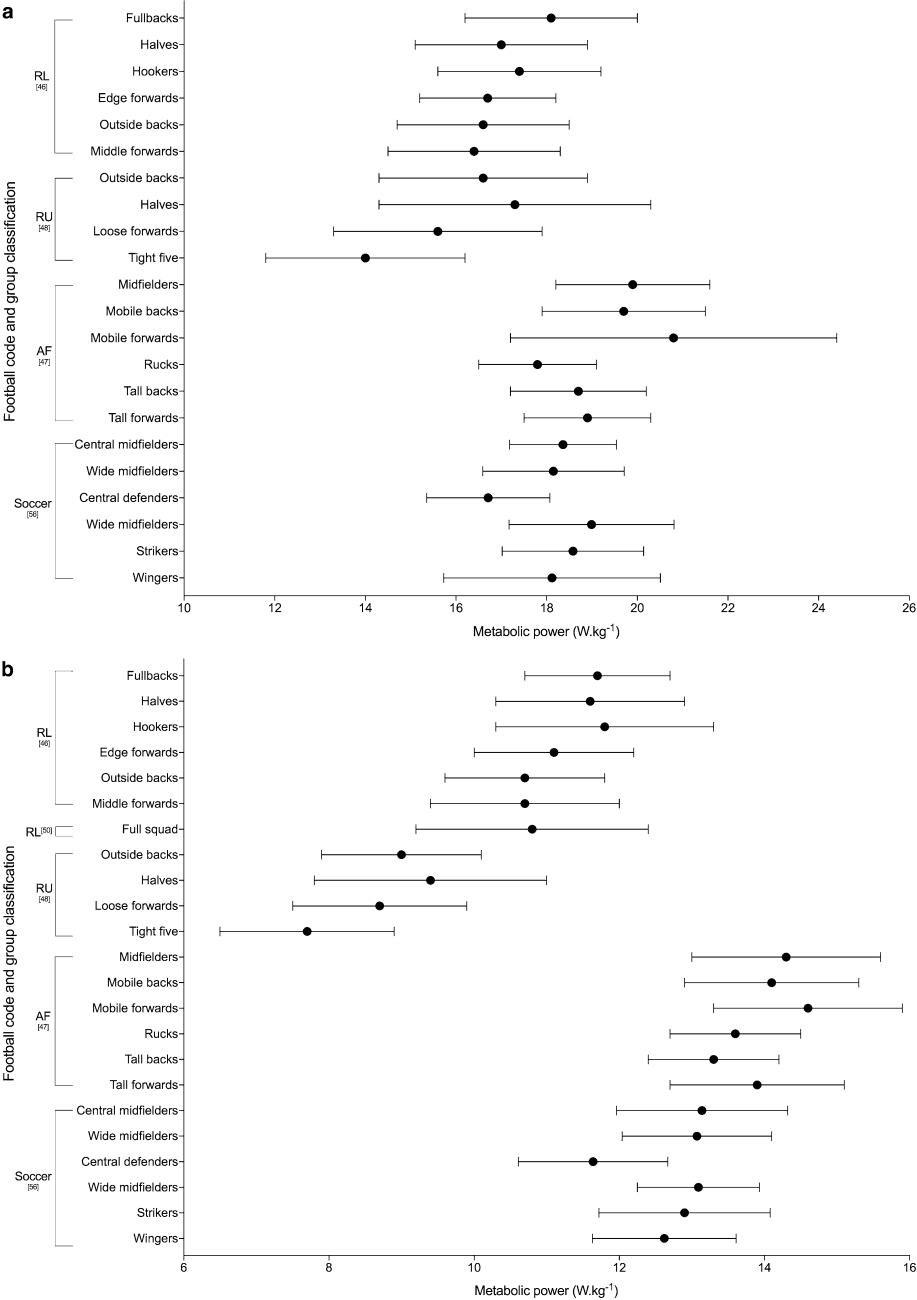

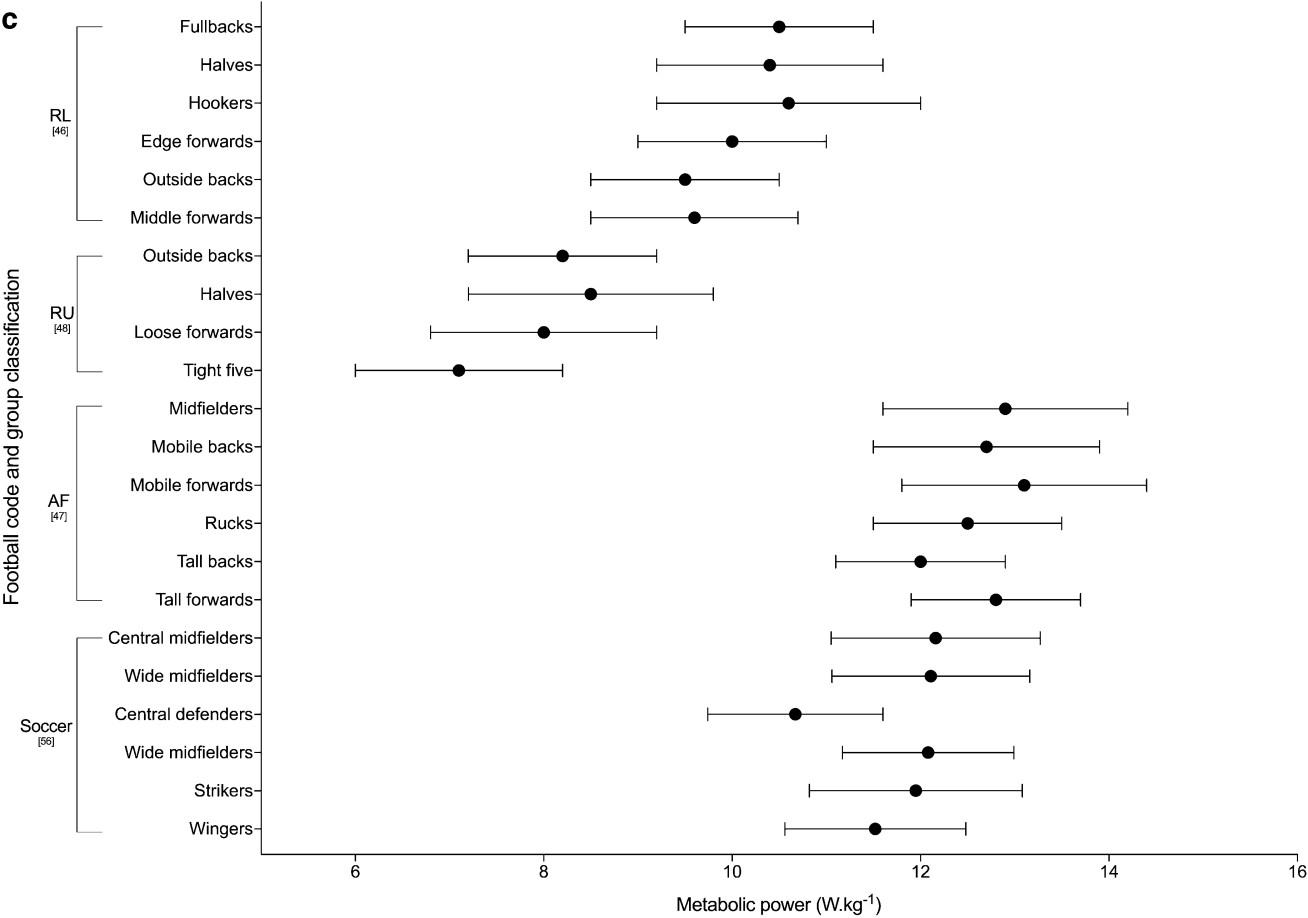
Duration-specific peak metabolic power (W·kg^−1^) in the football codes. **a** 1 min, **b** 5 min, **c** 10 min. Data are expressed as mean ± SD. *RL* rugby league, *RU* rugby union, *AF* Australian Football, *R7* rugby sevens

Metabolic power is based on a theoretical model that assumes accelerated running on flat terrain has the same energetic requirements as uphill running at a constant speed [64, 65]. It estimates the energetic cost of match play, using speed and acceleration derived from the microtechnology and aims to take into account the metabolically demanding movements of team sports that other variables may underestimate, i.e. accelerations and decelerations at low speeds, and high speed running [65]. Six of the studies included in the review used metabolic power to describe peak match demands [46–48, 50, 51, 56]. The peak 1-, 5- and 10-min metabolic power demands across the football codes are shown in Fig. 4. All other duration-specific periods are shown in Electronic Supplementary Material Fig. S3. Kempton et al. [50] reported distance covered over a ‘high power’ threshold set at > 20 W·kg^−1^ in rugby league, reporting the peak 5-min distance to be 185 m (95% CI 181–190). Carling et al. [54] used a surrogate measure of metabolic power and described high metabolic load (HLMD) as the distance covered at high intensity running (> 5.5 m·s^−2^) plus the distance covered while accelerating above 2 m·s^−2^. They reported the peak 5-min HMLD during rugby union match-play to be 30.6 ± 9.0 and 19.3 ± 4.9 m for backs and forwards, respectively [54].

#### Collisions

Collisions are a component of match-play in several of the football codes (e.g. rugby league, rugby union, rugby sevens, Australian Football); differences in the definition and classification of collisions exist across the codes, with some ambiguity of definitions within the codes [66]. Only two of the studies included in the review used microtechnology to quantify collisions [32, 49], both in rugby league and using segmental analysis, shown in Table 8. In rugby league collisions are defined as any tackles or hit-ups, and decoy runs or support runs where contact is made with a player in the defensive line [67]. Kempton et al. [44, 50] and Reardon et al. [53] also aimed to quantify peak collision demands, but used video analysis, thus are not considered in this review.

**Table 8:** Collisions, repeated high-intensity effort bouts, and PlayerLoad™ reported by the studies included in the review [32, 49, 57, 61]

Study	Sport	Level	Variable	Method of analysis	Time (min)	Collisions^a^ per minute	No. of collisions^a^	RHIE^b^ per minute	No. of RHIE^b^	PlayerLoad^c^	PlayerLoad^c^ per minute
Hulin et al. (2015) [32]	Rugby league	Professional	Adjustables high success^d^	Segmental	5	1.6 ± 0.5		0.35 ± 0.12			
			Adjustables low success^d^			1.1 ± 0.4		0.37 ± 0.18			
			Hit up forwards high success^d^			1.7 ± 0.5		0.37 ± 0.13			
			Hit up forwards low success^d^			1.7 ± 0.4		0.47 ± 0.11			
			Outside backs high success^d^			1.1 ± 0.3		0.24 ± 0.09			
			Outside backs low success^d^			0.8 ± 0.3		0.39 ± 0.09			
Hulin and Gabbett (2015) [49]	Rugby league	Semi-professional	Adjustables high success^d^	Segmental	5		6.3 ± 2.2		1.49 ± 0.76		
			Adjustables low success^d^				6.7 ± 1.8		1.40 ± 0.63		
			Hit up forwards high success^d^				7.1 ± 2.3		1.98 ± 0.91		
			Hit up forwards low success^d^				5.8 ± 1.8		1.94 ± 0.97		
			Outside backs high success^d^				6.8 ± 2.3		1.39 ± 0.74		
			Outside backs low success^d^				5.6 ± 1.8		1.24 ± 0.54		
Ramos et al. (2017) [57]	Soccer	International	Central defenders	Segmental	5					68 ± 12	
			Fullbacks							75 ± 5	
			Midfielders							70 ± 10	
			Forwards							70 ± 9	
Trewin et al. (2017) [61]	Soccer	International	Centre backs	Moving averages	5					70 ± 11	14.0 ± 2.1
			Fullbacks							71 ± 11	14.1 ± 2.3
			Midfielders							87 ± 16	17.5 ± 3.2
			Forwards							72 ± 16	14.3 ± 3.2
			Full squad							77 ± 16	15.3 ± 3.2

Data are expressed as mean ± SD

^a^*Collisions* any tackles or hit-ups, and decoy runs or support runs where contact is made with a player in the defensive line [67]

^b^*RHIE* repeated high-intensity effort bouts (three or more high-velocity, high-acceleration, or contact efforts with less than 21-s recovery between efforts [23, 32])

^c^*PlayerLoad*™ the accumulation of data from all axes (anteroposterior, mediolateral, and craniocaudal) [68]

^d^*High success* teams that won 71–75% of matches played. *Low success* teams that won 32–58% of matches played [32, 49]

#### Repeated High-Intensity Efforts

Repeated high-intensity effort bouts (RHIE) are defined as three or more high-velocity (> 5 m·s^−1^), high-acceleration (≥ 2.79 m·s^−2^) or contact efforts with less than 21 s recovery between efforts [23, 32]. Two studies reported the peak RHIE demands, either reporting as the absolute number or relative number [32, 49]; values are shown in Table 8.

#### PlayerLoad™

PlayerLoad™ is a manufacturer-specific parameter (Catapult Innovations, Melbourne, VIC, Australia) that provides a modified vector magnitude using accelerometer data, expressed in arbitrary units (AU) [68]. It is expressed through the accumulation of data from all axes (anteroposterior, mediolateral and craniocaudal) and is calculated as the square root of the sum of the squared instantaneous rate of change in acceleration in each of the three vectors divided by 100 [68]; it attempts to indicate the ‘total load’ experienced by the athlete. PlayerLoad™ was used by two studies to describe the peak 5 min during soccer match-play, using segmental [57] and moving averages [61] analysis; values are shown in Table 8.

#### Maximum Velocity

Reardon and colleagues [53] were the only investigators to use maximum velocity to describe the peak demands. They reported the maximum velocity obtained within the identified longest period of ball in play in rugby union; 4.9 (4.70–5.12) m·s^−1^ for tight five forwards, 5.72 (5.48–5.97) m·s^−1^ for back row forwards, 6.02 (5.79–6.25) m·s^−1^ for inside backs and 6.84 (6.57–7.12) m·s^−1^ for outside backs.

### Peak Demands in Football Codes

#### Rugby League

The peak match demands of rugby league were analysed by 26% (*n* = 7) of the studies included in the review, making it the most commonly investigated football code. Duration-specific peak relative distance values are shown in Fig. 2; peak 1-min relative distance ranged from ~ 154 to 179 m·min^−1^ across positional groups [36, 46, 63], and peak 5-min values ranged from ~ 92 to 126 m·min^−1^ [32, 36, 46, 49, 63]. The two studies that investigated position-specific running intensities reported peak relative distances for 1, 5 and 10 min to be the greatest for fullbacks (~ 172–179, ~ 118–122 and ~ 105–109 m·min^−1^ respectively), and lowest for the middle forwards (~ 154–163, ~ 102–111 and ~ 90–98 m·min^−1^, respectively) [36, 46]. Total or relative high speed running distance reported by the studies included in the review in rugby league are shown in Tables 6 and 7. Two studies [32, 49] used > 5 m·s^−1^ as the specified threshold for ‘high-speed running’; their data showed greater ranges in relative high-speed running distance for the professional clubs across the ‘high’ and ‘low’ success clubs match-play (~ 8–22 m·min^−1^) [32] than at the semi-professional level (~ 10–15 m·min^−1^) [49]. The highest relative high-speed running was reported for ‘low success’ professional outside backs (22 ± 2 m·min^−1^), and lowest for the professional ‘high-success’ adjustables (8 ± 5 m·min^−1^) [32, 49]. Other variables used to describe the peak demands in rugby league included AveAcc (Fig. 3 and Electronic Supplementary Material Fig. S2), metabolic power (Fig. 4 and Electronic Supplementary Material Fig. S3), collisions and RHIE (Table 8). Peak 1- and 5-min AveAcc ranged from ~ 1.22 to 1.28 and ~ 0.80 to 0.91 m·s^−2^, respectively, across positions, and was greatest for hookers at most duration-specific periods [46]. Peak 1- and 5-min metabolic power ranged from ~ 16.4 to 18.1 and ~ 10.7 to 11.7 W·kg^−1^, respectively, across positions [46].

#### Rugby Sevens

Peak 1-min total and high-speed distance covered in international level rugby sevens match play was reported by Murray and Varley [35] to be 183 ± 30 and 86 ± 30 m, respectively. Granatelli et al. [52] reported 31.2% of distance covered in 1 min to be the peak high-speed running (> 3.9 m·s^−1^) demands of professional level match-play. Electronic Supplementary Material Fig. S3 shows peak metabolic power for a 2-min duration [51]. Murray and Varley [35] used a moving average approach to identify peak acceleration count (≥ 2.87 m·s^−2^), reporting values of 3.8 ± 1.6 for a 1-min period.

#### Rugby Union

AveAcc (Fig. 3 and Electronic Supplementary Material Fig. S2) and metabolic power (Fig. 4 and Electronic Supplementary Material Fig. S3) were analysed by Delaney and colleagues [48] for different positional groups: outside backs, half backs, tight five and loose forwards. For 1-min durations peak AveAcc ranged from ~ 0.87 to 1.01 m·s^−2^ and metabolic power ranged from ~ 14.0 to 17.3 W·kg^−1^ across positional groups. Duration-specific peak relative distances were reported for different positional groups by three studies [48, 55, 58], shown in Fig. 2. For the peak relative distance values ranged from ~ 139 to 185 m·min^−1^ for the 1-min periods and from ~ 86 to 116 m·min^−1^ for the peak 5-min periods [48, 55, 58], with the highest demands reported for Academy level scrum halves at both durations [58]. Reardon et al. [53] identified the average longest period of ball in play for positional groups; tight five forwards (161 s), back row forwards (152 s), inside backs (154 s) and outside backs (155 s). The relative distances covered in these periods were: 109 (104–114) m·min^−1^ for tight five forwards, 111 (105–117) m·min^−1^ for back row forwards, 123 (117–129) m·min^−1^ for inside backs, and 124 (117–131) m·min^−1^ for outside backs. Total distances covered during the longest period of ball in play are shown in Table 5. Two studies reported relative high-speed running distance during rugby union match play (shown in Table 7), both of which reported greater distances for backs compared to forwards [53, 55]. Reardon et al. [53] also reported the number of ‘sprint’ efforts during the peak period: 0.02 (− 0.04 to 0.07) for tight five forwards, 0.02 (− 0.04 to 0.08) for back row forwards, 0.06 (0.00 to 0.11) for inside backs and 0.11 (0.04 to 0.16) for outside backs in the longest period of ball in play.

#### Soccer

Soccer is the only football code in the studies included in this review to report peak demands for female players during match play [57, 61]. Peak relative distance has been reported for different positional groups in male and female soccer over a number of duration specific periods (Fig. 2 and Electronic Supplementary Material Fig. S1). The 5-min duration values reported ranged from ~ 129 to 148 m·min^−1^ for male soccer [56] and from ~ 132 to 146 m·min^−1^ for female soccer [61] using the moving averages method of analysis, with the greatest values reported for the central midfielders and midfielders, respectively. Conversely, Ramos et al. [57] reported female ‘midfielders’ to cover the least total distance during the peak 5 min using segmental analysis, and fullbacks to cover the most (595 ± 51 vs. 653 ± 41 m, respectively) (Table 5). Five out of the six studies on soccer reported relative or total high-speed running, all of which used different thresholds, shown in Tables 6 and 7, respectively. Varley et al. [45] revealed greater high intensity running (> 4.17 m·s^−1^) distance covered using moving averages compared to segmental analysis (177 ± 91 vs. 142 ± 24 m) for peak 5-min periods of match play for male players. For female soccer players both studies reported fullbacks to cover the greatest high-speed running distance (~ 100–153 m), and centre backs the least (~ 67–101 m), over a 5-min epoch [57, 61]. Ramos et al. [57] also reported the peak 5-min ‘sprint’ distance (> 5.6 m·s^−1^) during female soccer match play: 37.1 ± 15.3 m for centre backs, 57.4 ± 16.9 m for fullbacks, 36.4 ± 13.6 m for midfielders and 60.7 ± 14.6 m for forwards. Metabolic power (Fig. 4 and Electronic Supplementary Material Fig. S3) and AveAcc (Fig. 3 and Electronic Supplementary Material Fig. S2) were reported over a range of duration-specific periods for male players during match play [56]. For 1-min durations peak AveAcc ranged from ~ 0.81 to 0.89 m·s^−2^ and ~ 16.7 to 19.0 W·kg^−1^ across positional groups [56]. Peak 5-min acceleration count of female match play was reported by two studies [57, 61], and deceleration count by one [57]. Ramos et al. [57] reported the lowest acceleration count for centre backs (2.11 ± 0.60 m·s^−2^), and highest for forwards (3.44 ± 1.13 m·s^−2^), whereas Trewin et al. [61] reported the highest count to be for both centre backs (3.44 ± 0.59 m·s^−2^) and forwards (3.44 ± 0.74 m·s^−2^). PlayerLoad™ was reported by two studies for peak 5-min periods (values are shown in Table 8). Using segmental analysis across positional groups, peak 5-min PlayerLoad™ values of ~ 68–75 AU were reported [57], compared to ~ 70–87 AU when moving averages analysis was used [61].

#### Australian Football

Figure 2 and Table 6 show duration-specific peak relative distance and relative high-speed running distance, respectively, for durations from 1 to 10 min. Relative distance ranged from ~ 199 to 215 m·min^−1^ for peak 1-min durations, and from ~ 131 to 141 m·min^−1^ for 10 min across positional groups, with higher intensities for midfielders and mobile forwards than tall backs [47]. The highest relative high-speed running distance was reported for mobile forwards, and lowest for rucks, across all durations investigated [47]. Duration-specific peak AveAcc and metabolic power for 1-, 5- and 10-min durations are shown in Figs. 3 and 4, respectively, with peak 1-min values ranging from ~ 0.94 to 1.05 m·s^−2^ for AveAcc and ~ 17.8 to 20.8 W·kg^−1^ for metabolic power, across positional groups. At durations of 1, 5 and 10 min mobile backs were reported to have the greatest AveAcc (1.05 ± 0.17, 0.81 ± 0.13 and 0.72 ± 0.05 m·s^−2^, respectively), and mobile forwards the greatest metabolic power (20.8 ± 3.6, 14.6 ± 1.3 and 12.8 ± 1.3 W·kg^−1^, respectively) [47].

#### Gaelic Football

Relative total, high-speed running and sprint distances were reported by Malone et al. [62] for 1–10 min across different positions: full-back, half-back, midfield, half-forward and full-forward. For the 1-min duration peak relative distance ranged from ~ 194 to 255 m·min^−1^ and relative high-speed running distance ranged from ~ 36 to 50 m·min^−1^ across positions, with midfielders reported to cover the greatest and full-backs and full-forwards the least for both variables (Fig. 2). The peak acceleration distance covered over 5 min was reported by two studies [59, 60]. A whole squad average peak acceleration distance of 296 ± 10 m was reported in one study [59], but position-specific values have also been reported [60]: 372 ± 107 m for full-backs, 458 ± 79 m for half-backs, 538 ± 58 m for midfielders and 455 ± 95 m for half-forwards, 349 ± 98 m for full-forwards.

## Discussion

This is the first review to summarise the use of microtechnology to quantify the peak match demands of the football codes. Following the screening process, 27 studies were identified that have used microtechnology to determine the peak-match demands in one of the football codes. The use of microtechnology to identify peak-match demands appears to have increased over recent years, with the earliest identified study published in 2012 [45]. Studies were identified in six codes: soccer [40, 42, 45, 56, 57, 61], rugby union [48, 53–55, 58], rugby sevens [35, 41, 51, 52], rugby league [32, 36, 44, 46, 49, 50, 63], Australian Football [25, 47] and Gaelic Football [59, 60, 62]. There is a bias towards research in male athletes; only two of the studies included in the review investigated female match-play, both in soccer [57, 61].

### Methodology Used to Quantify Peak Match Demands

Three different methodologies were identified in this review: segmental analysis, moving averages and the longest period of ball in play. Early research predominately used segmental analysis, whilst later studies largely used the moving averages approach. Moving averages was the most commonly used method and was used by at least one study on each of the football codes.

The use of moving averages over segmental analysis to identify the peak demands is supported by this review. Two studies directly compared segmental and moving averages to identify the superior method for quantifying peak demands [45, 55]. Varley et al. [45] found the distance covered at high speed to be 25% higher in the peak 5 min of soccer match-play when using moving averages (segmental vs. moving averages: 142 ± 24 vs. 177 ± 91 m). Similarly, Cunningham et al. [55] reported relative total and high-speed running distance to be ~ 11–20% higher across epochs of 60–300 s when using moving averages compared to segmental analysis in rugby union match play. Sparks and colleagues [42] also showed higher values for the peak 5-min relative high-speed running identified via moving averages than segmental, at all activity level groups (high: ~ 59 vs. ~ 36 m·min^−1^, moderate: ~ 49 vs. 28 m·min^−1^, low: ~ 38 vs. 20 m·min^−1^) in soccer. Additionally, 1-mine peak running demands in rugby sevens match play reported using moving averages were ~ 183 m·min^−1^ [35], compared to ~ 123–130 m·min^−1^ found using segmental analysis [52]. The superiority of moving averages is due to its ability to capture the fluctuations in demands that may be missed with the use of segmental analysis. For example, if the peak demands occur between 3 and 7 min, segmental analysis that takes averages from 0–5 and 5–10 min would miss the full peak period and consequently underestimate the demands. Studies that report higher peak values using segmental analysis are likely due to the demands of the cohort investigated as opposed to the method used. For example, Hulin et al. [32] reported higher values for the peak 5-min periods using segmental analysis during rugby league match-play. But these values are only for the ‘low-success’ teams, which could be explained by the higher running demands reported when defending [69]. However, despite moving averages being the recommended methodology for identifying the peak match demands using microtechnology, it requires more experienced personnel to undertake the analysis and likely more time as the raw velocity files are currently required to be exported and analysed in customised software (e.g. R). Therefore, to increase the suitability of such a method of analysis in practice companies should consider the inclusion of the ability to generate moving averages of user-specified durations in the analysis software.

In addition to different methodologies, the review has identified a range of durations used to identify peak-match demands (10 s to 10 min). It is evident that the longer the duration of the peak period, the lower the intensity [36, 46–48], which is due to the physiological, contextual and technical-tactical demands of the sport. As the peak duration-specific periods increase, players will be unable to physiologically maintain the same intensity, due to the shift in the energy continuum [70]. Additionally, Duthie et al. [71] showed that the physiological capabilities of individual athletes will influence the magnitude of the decrease in intensity. As the duration increases, faster and stronger athletes will experience a greater decrease in running intensity [55]. However, it is unlikely that the physiological demands are the primary reason for the decline in intensity considering the low average running speeds for the peak periods identified in this review. For example, the highest peak 1-min running demands identified in rugby union were ~ 185 m·min^−1^, equating to ~ 3.0 m·s^−1^, which were lower than ‘high-speed running’ thresholds and only ~ 59% of the final velocity achieved during the 30-15 Intermittent Fitness Test in professional rugby union players [72]. It could therefore be suggested that the main reason for the decline in intensity is contextual, as a consequence of the technical-tactical demands of the football codes [71, 73]. The longer the period investigated, the more likely there is to be a stoppage in play due to an error being made, a score, a stoppage of time by the referee or the ball going out of play, thus reducing the need for players to maintain a certain intensity. For example, in semi-professional rugby league, 41 ± 6 stoppages in play have been reported to occur during matches [74], with the most common reasons being for scrums, penalties and tries [75]. Furthermore, it has been reported that senior professional match-play (National Rugby League) demonstrates longer periods of ball-in-play periods, and a smaller proportion of short duration activity cycles than during junior professional matches (National Youth Competition) [75], thus suggesting the decline in intensity will also be impacted by the level of play investigated.

Only two of the studies included in the review identified peak demands shorter than 1 min [58, 63]. However, considering the difference in the physiological demands of short and long peak periods [76], and likely difference in technical-tactical demands, it is important that both short and longer duration periods of play are identified. The different duration-specific intensities can be utilised in different ways with different durations emphasising different priorities between physiological preparation and tactical-technical ability. For example, short windows of 30 s could be used for running conditioning drills with repeated exposure, and the peak 10 min for monitoring coach led drills to replicate the intensity of game play while the focus is on technical-tactical ability. Further consideration should be placed on interchange players, especially in the codes where ‘rolling’ substitutions are permitted; for players on the field for less than 10 min, duration-specific intensities of shorter durations are more important. It is evident that for both research and practice, consideration should be taken over the duration of the window used to identify the peak match demands.

### Variables Used to Analyse Peak Match Demands

The most commonly used variables identified in this review were velocity-based running variables. Whilst peak-running demands are valuable the velocity-based variables alone can underestimate the internal load placed upon the players, not counting metabolically demanding movements such as accelerations/decelerations and collisions [64]. To overcome this, several studies have included the use of acceleration and deceleration variables and metabolic power [35, 40, 46–48, 50, 51, 56, 57, 59–61]. The most valid acceleration/deceleration variable is reported to be AveAcc [77]. However, the difficulty for coaches to conceptualise AveAcc or metabolic power, and then manipulate training drill content from such values, limits their application in practice to inform day-to-day training prescription. Nevertheless, the quantification of the metabolic cost of the movements that players undertake is important, and despite questions around the validity of metabolic power [78], it is currently the best proxy measure to incorporate the physiological demands of constant and accelerated demands. But in contact-based sports consideration of the collisions is also required.

Most of the studies included in the review used multiple variables to assess the duration-specific peak-match demands [25, 32, 35, 46–52], identifying the peak period for each variable as separate constructs (i.e. what is the peak 10-min period for relative distance covered, and what is the peak period for AveAcc). Although this approach is useful for detecting the ‘worst-case scenario’ for individual variables, and thus prescription of specific training, it is likely that determining the interaction between all external load demands that occur during predefined periods would be beneficial. Examples would be, on the one hand, determining duration-specific peak running demands then identifying the number of collisions and or acceleration/decelerations that occur during this period and, on the other hand, establishing the peak collision demands and then identifying the associated relative distances during this period. This would provide practitioners with more useful information to aid in the prescription and monitoring of training drills, ensuring players are exposed to the peak-running demands alongside other stimuli that occur during match-play at the appropriate playing level. Whilst the addition of other variables would enhance the usefulness of the peak running demands, the common use of ‘live’ feedback during skills must be considered. Not all variables are available to every GPS consumer, nor have they been deemed valid and reliable for live monitoring (e.g. collisions and AveAcc).

### Summary of Peak Match Demands Across the Football Codes

Of the six football codes assessed in this review, Gaelic Football appeared to have the highest peak-running demands at all durations, followed by Australian Football. For example, in Gaelic Football match-play peak 1-min relative distance ranged from ~ 194 to 255 m·min^−1^ [62] compared to ~ 199 to 223 m·min^−1^ for Australian Football [47], ~ 178 to 202 m·min^−1^ for soccer [56], ~ 139 to 185 m·min^−1^ for rugby union [48, 55, 58], ~ 123 to 183 m·min^−1^ for rugby sevens [35, 52] and ~ 154 to 179 m·min^−1^ for rugby league [36, 46]. Similarly, for peak 5 min, Gaelic Football match-play reported higher relative distances than the other codes: ~ 155–186 m·min^−1^ for Gaelic Football [62], ~ 153–160 m·min^−1^ for Australian Football [47], ~ 99–148 m·min^−1^ for soccer [42, 56, 61], ~ 92–126 m·min^−1^ for rugby league [32, 36, 46, 49, 63] and ~ 86–116 m·min^−1^ for rugby union [48]. Australian Football match-play has previously been reported to have higher whole-match running demands, with significantly higher average match intensities and high-velocity running compared to soccer and rugby league [22]. Although Australian Football appeared to have one of the highest velocity-based running demands, all duration-specific AveAcc values reported were highest in rugby league. For example, the peak 1 min in rugby league was ~ 1.22–1.28 m·s^−2^ [46] compared to ~ 0.94–1.05 m·s^−2^ in AFL [47] and ~ 0.87–1.01 m·s^−2^ in rugby union [48]. Therefore, prescription of training based on peak demands should be specific to the football code of interest.

The differences in peak demands between the codes are likely due to the regulations and tactical demands of the codes. The three football codes with the highest velocity-based running demands (Gaelic Football, Australian Football and soccer) are all ‘360-degree’ sports (i.e. the ball can be passed in any direction), permitting more movement on and off the ball. Conversely, movement in the rugby codes is limited through them being ‘180 degrees’ by nature (i.e. the ball can only be passed backwards), and the presence of the defensive line in front of the attacking play. The larger pitch size in Gaelic and Australian Football compared to soccer and the rugby codes would allow greater opportunities for space and thus greater distance to be covered [79, 80]. Additionally, the 90 ‘rolling’ interchanges permitted in the Australian Football compared to the limit of three substitutions in soccer can impact on the peak running demands through fatigue and potential pacing strategies [37, 81]. The presence of collisions will also influence the peak demands, with higher collision counts likely resulting in lower velocity-based running demands [82, 83], but conversely higher acceleration/deceleration demands [82]. In addition, the 10-m ‘on-side’ rule that separates the attacking and defensive teams in rugby league likely explains the higher acceleration/ deceleration demands observed [84].

### Limitations

A limitation of the current literature on the peak match demands in the football codes is the lack of studies that use multiple clubs from the respective competition. Individual teams may use certain tactics that influence their match demands, thus reducing the generalisability of the findings reported to other teams. Additionally, 12 out of the 16 studies in this review that investigated external load in speed zones used different thresholds to classify ‘high-speed running’. The lack of homogeneity of speed thresholds used across the studies limits comparison of high-speed running between codes, levels and age groups. The different hardware and firmware of the microtechnology used to collect data as well as the different software used to analyse the data pose further issues with comparisons due to the differences in data collection methods (i.e. Doppler-shift vs. positional differentiation) and data processing (i.e. algorithms used to smooth data) [7]. A limitation at the review level exists around the ability to summarise the peak demands across the football codes due to the different methodologies, cohorts and positional groups investigated. The lack of consistency of methods, positional groups, speed thresholds and variables used prevented a meta-analysis being carried out. This would be beneficial for practitioners, providing normative values for the peak demands of the football codes at different age groups and levels of competition.

### Future Directions

Further studies using moving averages with multiple clubs are required to provide more generalisable peak match demands across the different football codes for specific levels of play. Research identifying other external demands during duration-specific peak running demands, such as collision count, would further enhance exercise prescription as well as provide a more in-depth comparison between the levels of play. Additionally, knowledge of when the peak match demands occur, through time-stamps from the microtechnology alongside video analysis, would provide coaches with information on the technical-tactical demands during these periods alongside the physical demands. More studies that identify other physical demands (e.g. collisions and accelerations), in addition to the locomotive variables, as well as technical-tactical demands, over both shorter (i.e. 10 and 30 s) and longer duration-specific periods (i.e. 10 and 30 s) are required to aid in prescribing more specific training drills. Finally, current peak-demands research is focused only on the external load encountered by players with no consideration of the associated internal load, or the peak internal load encountered. Microtechnology now has the capability to collect and process heart-rate data and therefore could be used to investigate this. However, this is still difficult in the rugby codes during match-play due to the contact demands and practical issues with the hardware. Finally, for a meta-analysis of peak demands to be conducted more consistency is required across the methodologies of match demands research. For example, with a uniform threshold used for high-speed running it is not possible to compare the demands between levels and ages within individual football codes, let alone compare between codes.

## Conclusion

The quantification of the peak match demands across the football codes is important to appropriately prepare players for the most intense periods of match-play. This review has identified several methods using microtechnology to quantify the peak match demands of the football codes: moving averages, segmental and longest period of ball in play. The moving averages method is deemed the superior method of analysis, but requires greater analytical skills and time to analyse. Multiple durations and variables are used in current research, all of which could be deemed relevant; thus, practitioners and researchers should choose durations and variables specific to their needs. This review has revealed code-specific peak match demands that can be used by practitioners for the prescription of conditioning drills and monitoring of training intensity. However, current research is limited by the high number of one-club studies as well as the lack of shorter duration-specific periods, and further research is required.

## Electronic supplementary material

Below is the link to the electronic supplementary material.

Figure S1. Duration-specific peak relative distance (m·min^-1^) in the football codes. a = 10-, 15- and 30-seconds, b = 2-minutes, c = 3-minutes, d = 4-minutes, e = 6-minutes, f = 7-minutes, g = 8-minutes, h = 9-minutes. Data expressed as mean ± SD. RL = rugby league, RU = rugby union, AF = Australian Football, R7 = rugby sevens, GF = Gaelic Football

Figure S2. Duration specific peak average absolute acceleration/deceleration (AveAcc; m·s^-2^) in the football codes. a = 2-minutes, b = 3-minutes, c = 4-minutes, d = 6-minutes, e = 7-minutes, f = 8-minutes, g = 9-minutes. Data expressed as mean ± SD. RL = rugby league, RU = rugby union, AF = Australian Football

Figure S3. Duration-specific peak metabolic power (W·kg^-1^) in the football codes. a = 2-minutes, b = 3-minutes, c = 4-minutes, d = 6-minutes, e = 7-minutes, f = 8-minutes, g = 9-minutes. Data expressed as mean ± SD. RL = rugby league, RU = rugby union, AF = Australian Football, R7 = rugby sevens
