# Supplementary material for: The Use of Microtechnology to Quantify the Peak Match Demands of the Football Codes: A Systematic Review
Source: Sports Med. 2018 Aug 7;48(11):2549–75. doi: 10.1007/s40279-018-0965-6 (PMC6182461; doi:10.1007/s40279-018-0965-6)
Supplement: Supplementary file 1 — Figure S1. Duration-specific peak relative distance (m·min-1) in the football codes. a = 10-, 15- and 30-seconds, b = 2-minutes, c = 3-minutes, d = 4-minutes, e = 6-minutes, f = 7-minutes, g = 8-minutes, h = 9-minutes. Data expressed as mean ± SD. RL = rugby league, RU = rugby union, AF = Australian Football, R7 = rugby sevens, GF = Gaelic Football [file 40279_2018_965_MOESM1_ESM.pdf]

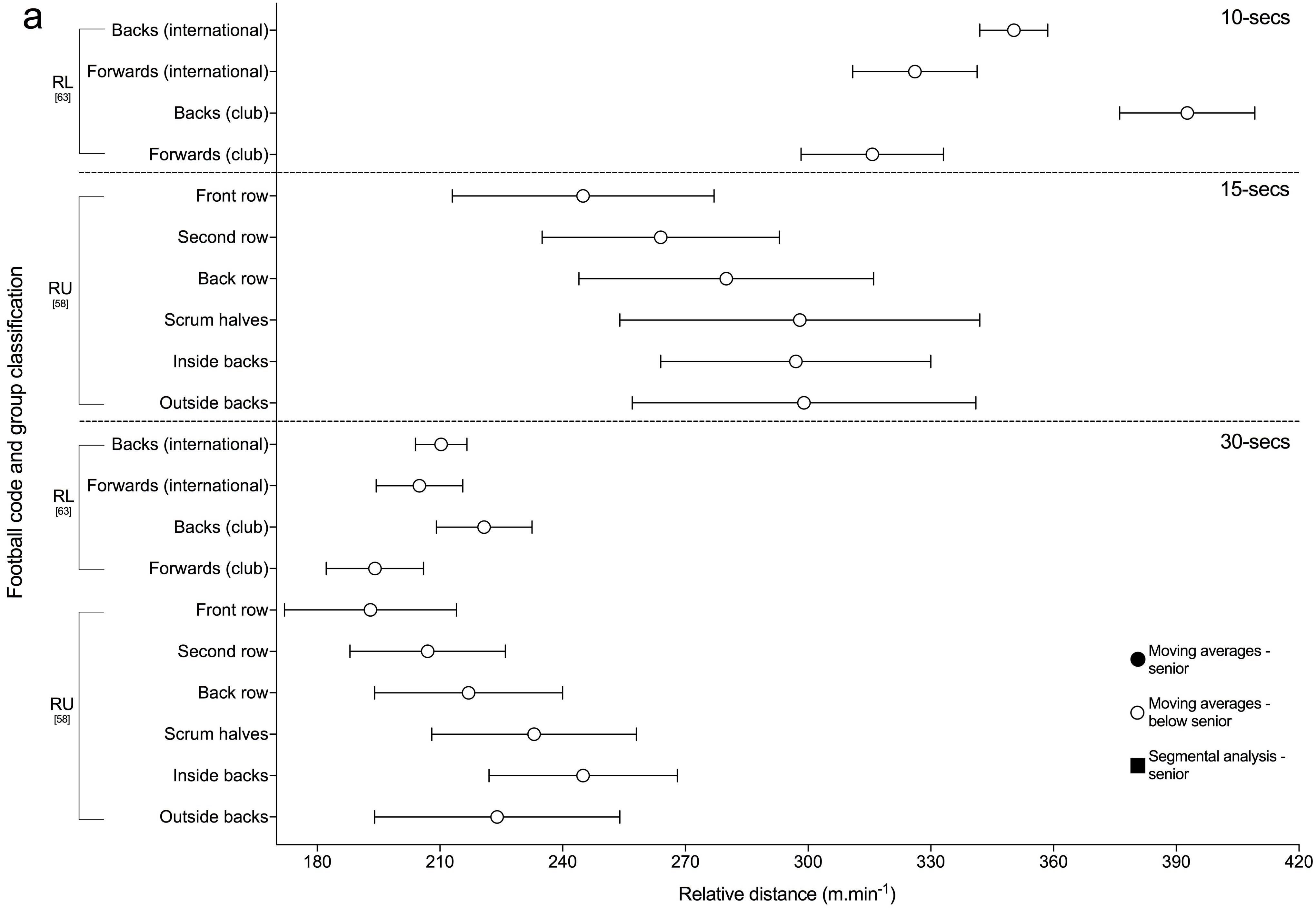

a
10-secs
15-secs
30-secs
RL
[63]
RU
[58]
Backs (international)
Forwards (international)
Backs (club)
Forwards (club)
Front row
Second row
Back row
Scrum halves
Inside backs
Outside backs
Football code and group classification
Relative distance (m.min-1)
180
210
240
270
300
330
360
390
420
Moving averages - senior
Moving averages - below senior
Segmental analysis - senior

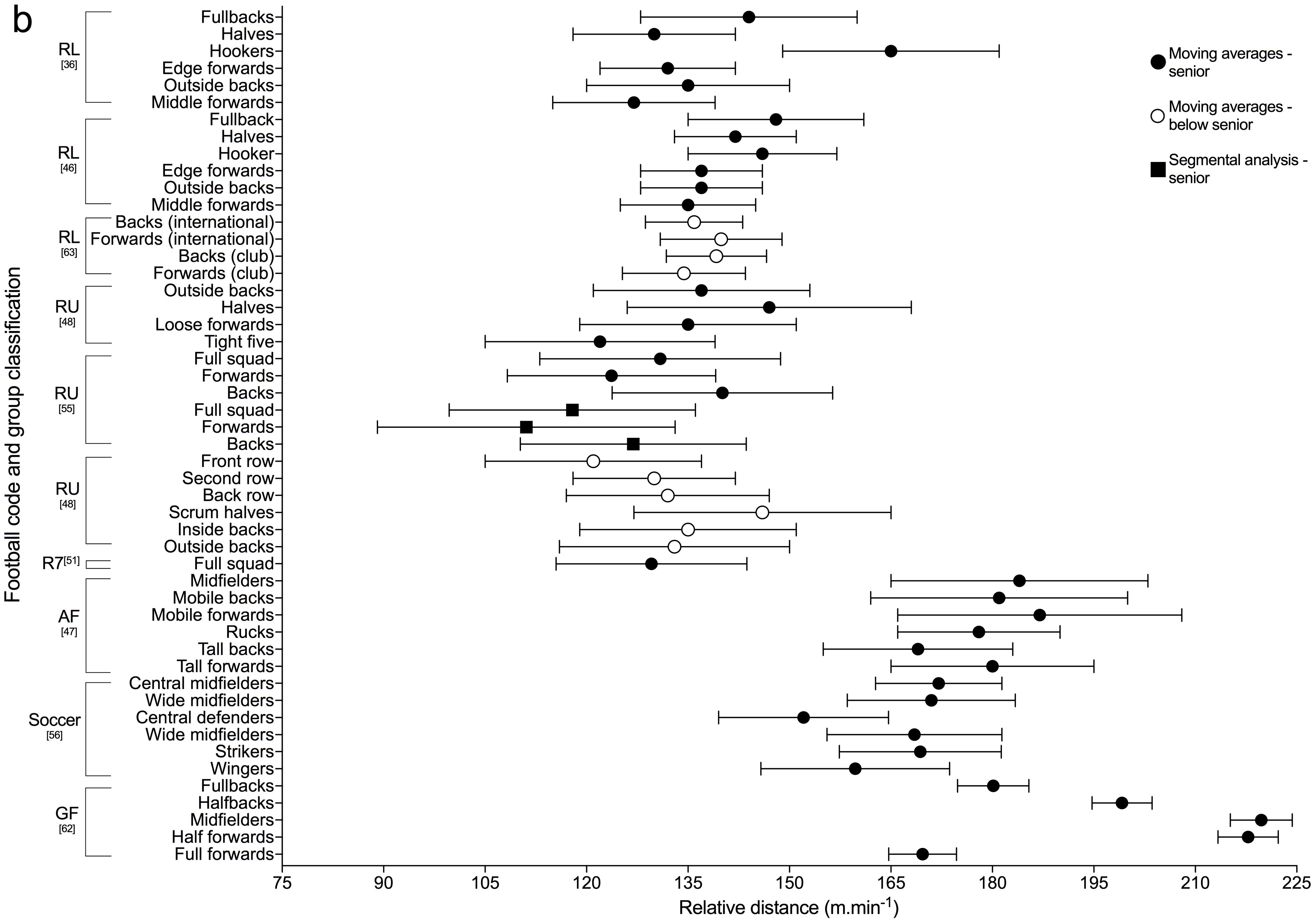

b
RL [36]
Fullbacks
Halves
Hookers
Edge forwards
Outside backs
Middle forwards
RL [46]
Fullback
Halves
Hooker
Edge forwards
Outside backs
Middle forwards
RL [63]
Backs (international)
Forwards (international)
Backs (club)
Forwards (club)
RU [48]
Outside backs
Halves
Loose forwards
Tight five
RU [55]
Full squad
Forwards
Backs
Full squad
Forwards
Backs
RU [48]
Front row
Second row
Back row
Scrum halves
Inside backs
Outside backs
R7 [51]
Full squad
AF [47]
Midfielders
Mobile backs
Mobile forwards
Rucks
Tall backs
Tall forwards
Soccer [56]
Central midfielders
Wide midfielders
Central defenders
Wide midfielders
Strikers
Wingers
GF [62]
Fullbacks
Halfbacks
Midfielders
Half forwards
Full forwards
Football code and group classification
75
90
105
120
135
150
165
180
195
210
225
Relative distance (m.min-1)
Moving averages - senior
Moving averages - below senior
Segmental analysis - senior

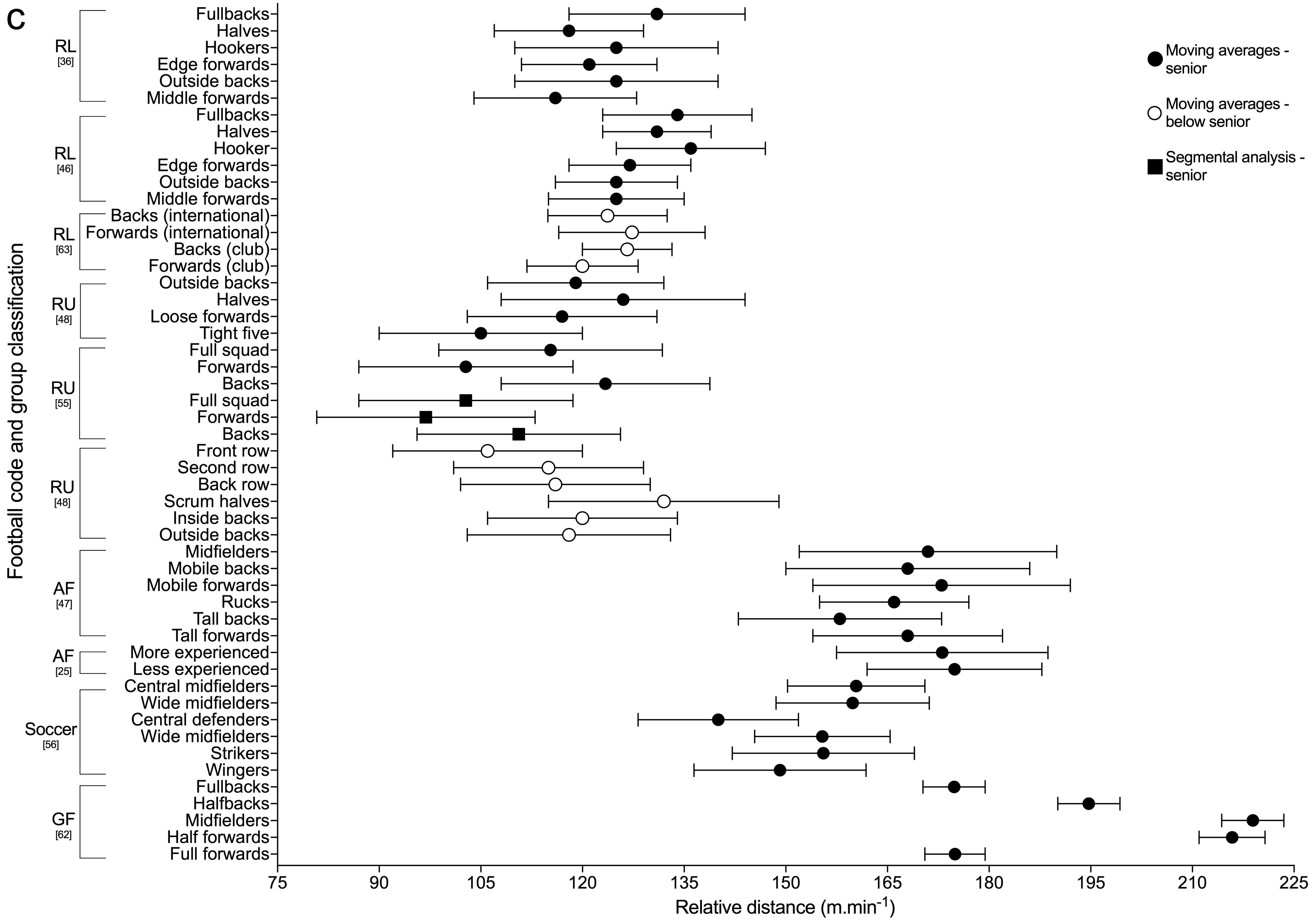
C
RL [36]
Fullbacks
Halves
Hookers
Edge forwards
Outside backs
Middle forwards
RL [46]
Fullbacks
Halves
Hooker
Edge forwards
Outside backs
Middle forwards
RL [63]
Backs (international)
Forwards (international)
Backs (club)
Forwards (club)
RU [48]
Outside backs
Halves
Loose forwards
Tight five
RU [55]
Full squad
Forwards
Backs
Full squad
Forwards
Backs
RU [48]
Front row
Second row
Back row
Scrum halves
Inside backs
Outside backs
AF [47]
Midfielders
Mobile backs
Mobile forwards
Rucks
Tall backs
Tall forwards
AF [25]
More experienced
Less experienced
Soccer [56]
Central midfielders
Wide midfielders
Central defenders
Wide midfielders
Strikers
Wingers
GF [62]
Fullbacks
Halfbacks
Midfielders
Half forwards
Full forwards
Football code and group classification
75 90 105 120 135 150 165 180 195 210 225
Relative distance (m.min-1)
Moving averages - senior
Moving averages - below senior
Segmental analysis - senior

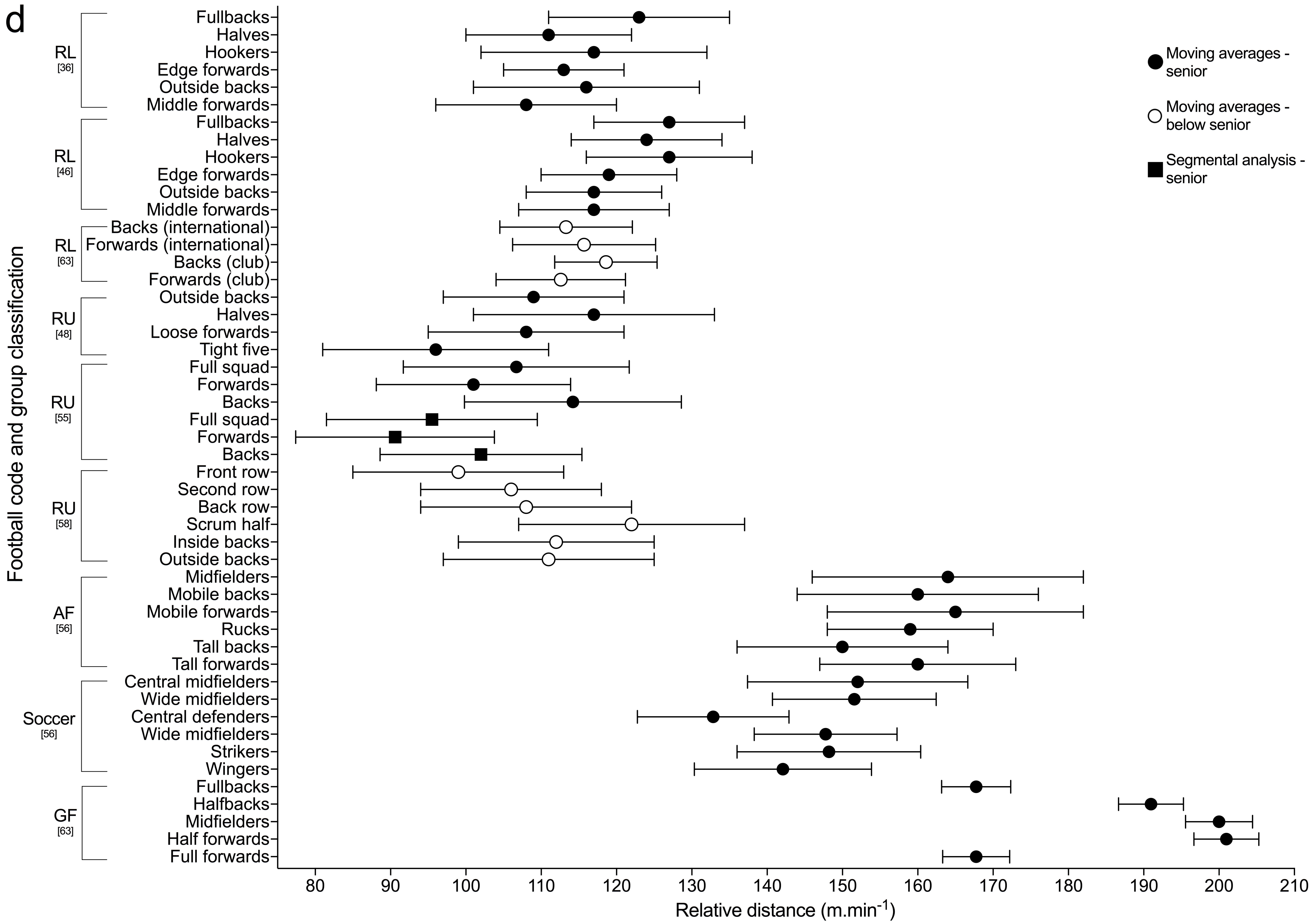

d
RL [36]
Fullbacks
Halves
Hookers
Edge forwards
Outside backs
Middle forwards
RL [46]
Fullbacks
Halves
Hookers
Edge forwards
Outside backs
Middle forwards
RL [63]
Backs (international)
Forwards (international)
Backs (club)
Forwards (club)
RU [48]
Outside backs
Halves
Loose forwards
Tight five
RU [55]
Full squad
Forwards
Backs
Full squad
Forwards
Backs
RU [58]
Front row
Second row
Back row
Scrum half
Inside backs
Outside backs
AF [56]
Midfielders
Mobile backs
Mobile forwards
Rucks
Tall backs
Tall forwards
Soccer [56]
Central midfielders
Wide midfielders
Central defenders
Wide midfielders
Strikers
Wingers
GF [63]
Fullbacks
Halfbacks
Midfielders
Half forwards
Full forwards
Football code and group classification
80 90 100 110 120 130 140 150 160 170 180 190 200 210
Relative distance (m.min-1)
Moving averages - senior
Moving averages - below senior
Segmental analysis - senior

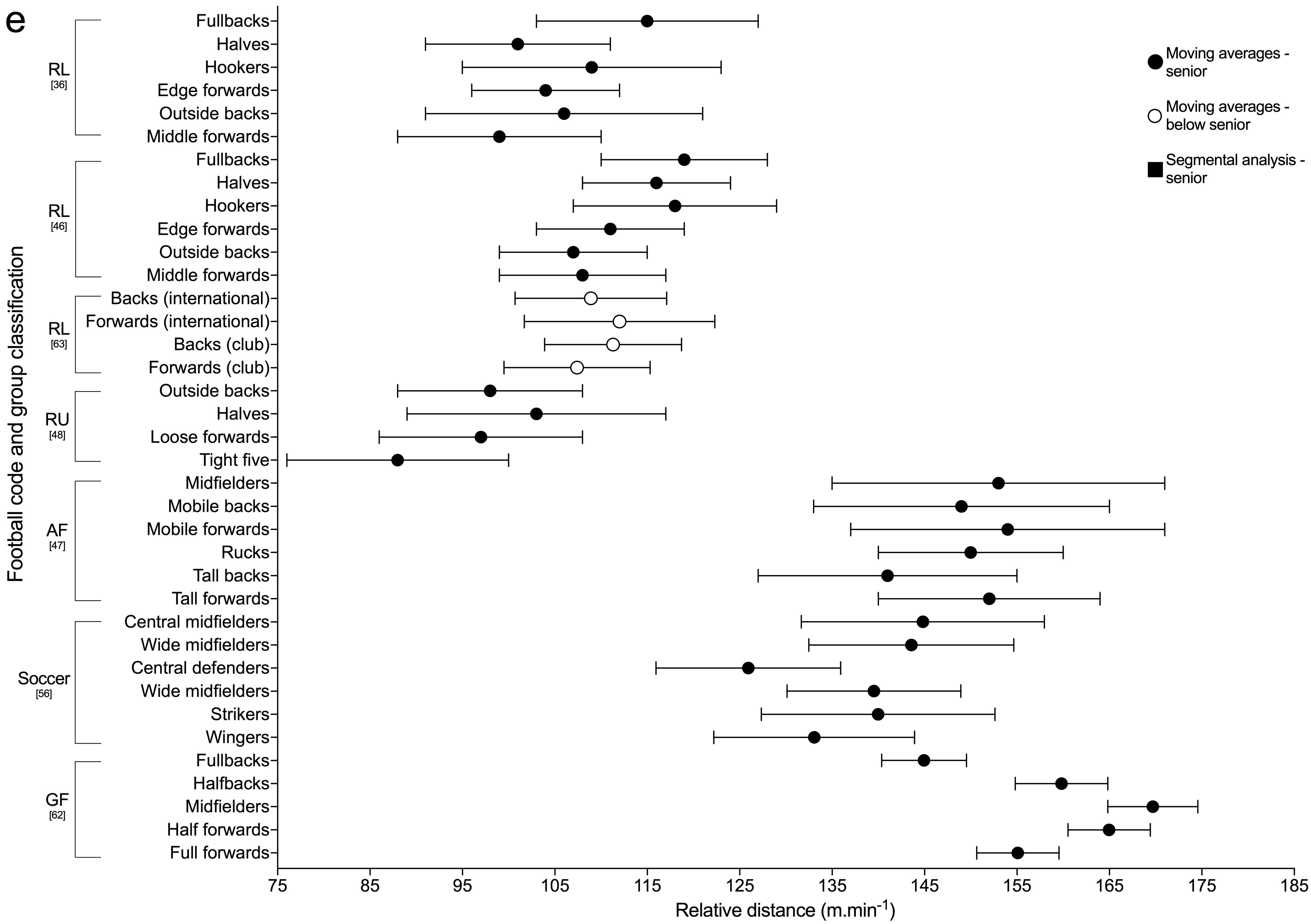

e
RL [36]
Fullbacks
Halves
Hookers
Edge forwards
Outside backs
Middle forwards
RL [46]
Fullbacks
Halves
Hookers
Edge forwards
Outside backs
Middle forwards
RL [63]
Backs (international)
Forwards (international)
Backs (club)
Forwards (club)
RU [48]
Outside backs
Halves
Loose forwards
Tight five
AF [47]
Midfielders
Mobile backs
Mobile forwards
Rucks
Tall backs
Tall forwards
Soccer [56]
Central midfielders
Wide midfielders
Central defenders
Wide midfielders
Strikers
Wingers
GF [62]
Fullbacks
Halfbacks
Midfielders
Half forwards
Full forwards
Football code and group classification
Moving averages - senior
Moving averages - below senior
Segmental analysis - senior
75
85
95
105
115
125
135
145
155
165
175
185
Relative distance (m.min-1)

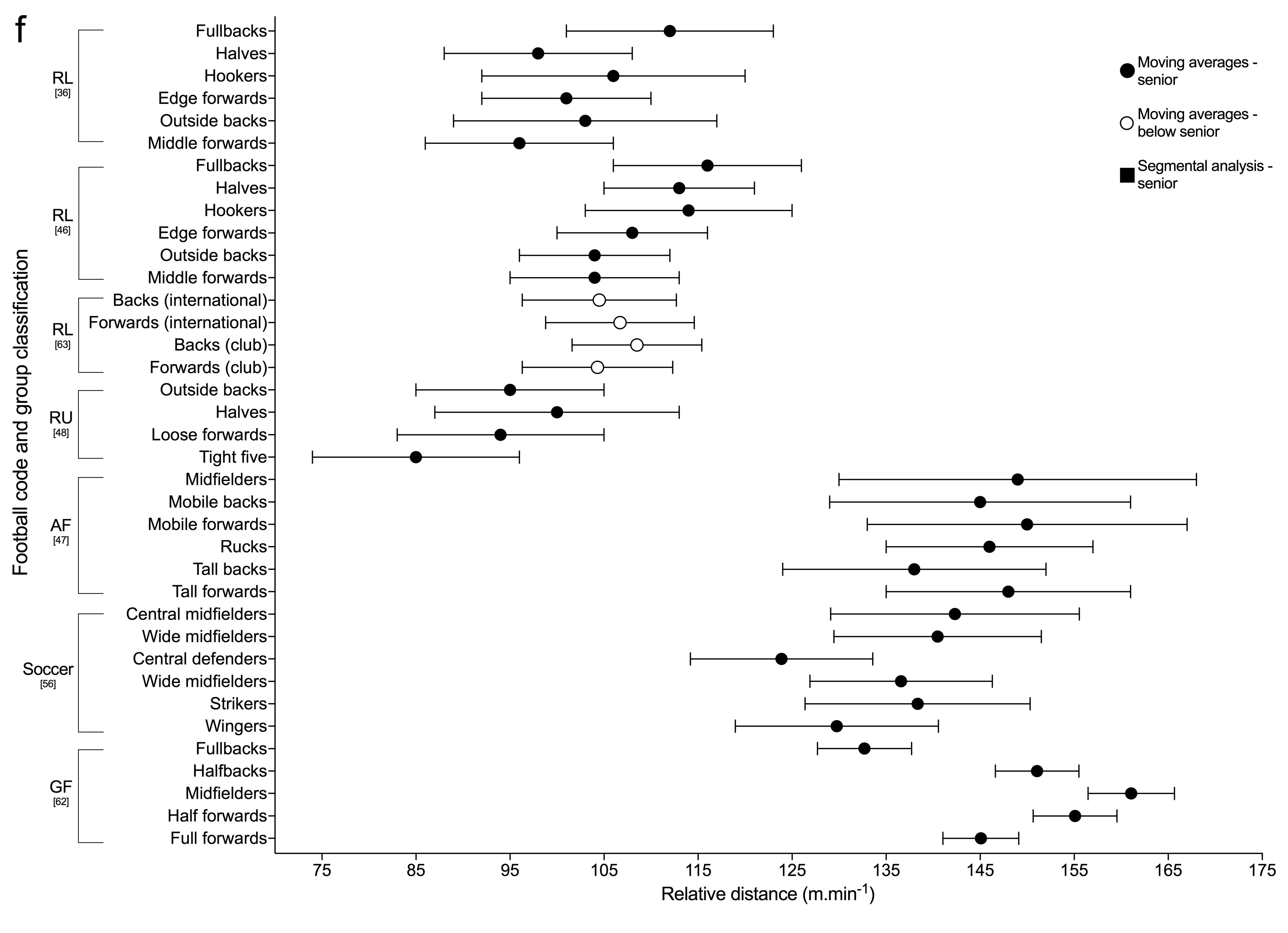

f
Moving averages - senior
Moving averages - below senior
Segmental analysis - senior
RL [36]
Fullbacks
Halves
Hookers
Edge forwards
Outside backs
Middle forwards
RL [46]
Fullbacks
Halves
Hookers
Edge forwards
Outside backs
Middle forwards
RL [63]
Backs (international)
Forwards (international)
Backs (club)
Forwards (club)
RU [48]
Outside backs
Halves
Loose forwards
Tight five
AF [47]
Midfielders
Mobile backs
Mobile forwards
Rucks
Tall backs
Tall forwards
Soccer [56]
Central midfielders
Wide midfielders
Central defenders
Wide midfielders
Strikers
Wingers
GF [62]
Fullbacks
Halfbacks
Midfielders
Half forwards
Full forwards
Football code and group classification
75 85 95 105 115 125 135 145 155 165 175
Relative distance (m.min-1)

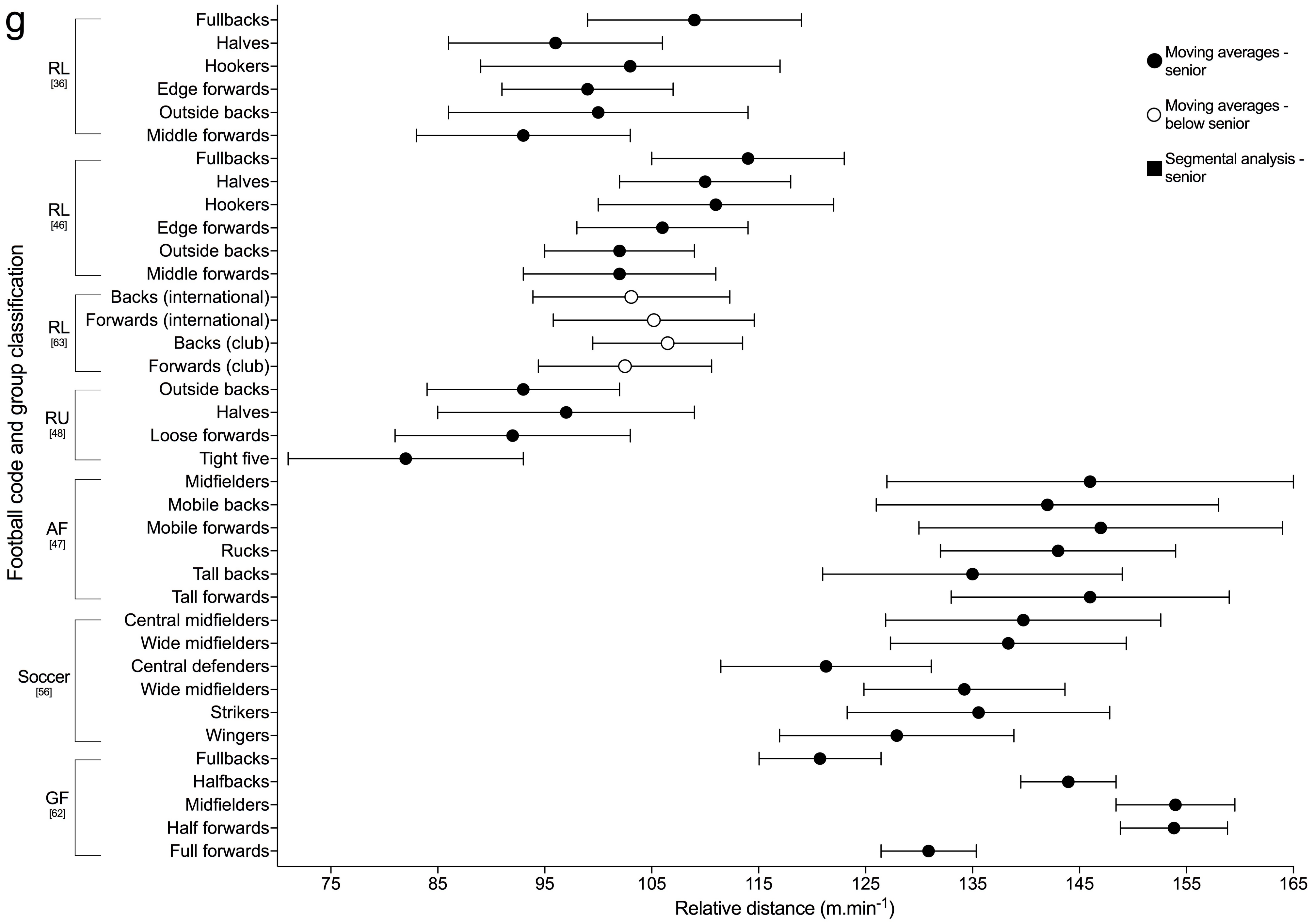
g
RL [36]
Fullbacks
Halves
Hookers
Edge forwards
Outside backs
Middle forwards
RL [46]
Fullbacks
Halves
Hookers
Edge forwards
Outside backs
Middle forwards
RL [63]
Backs (international)
Forwards (international)
Backs (club)
Forwards (club)
RU [48]
Outside backs
Halves
Loose forwards
Tight five
AF [47]
Midfielders
Mobile backs
Mobile forwards
Rucks
Tall backs
Tall forwards
Soccer [56]
Central midfielders
Wide midfielders
Central defenders
Wide midfielders
Strikers
Wingers
GF [62]
Fullbacks
Halfbacks
Midfielders
Half forwards
Full forwards
Football code and group classification
Moving averages - senior
Moving averages - below senior
Segmental analysis - senior
75
85
95
105
115
125
135
145
155
165
Relative distance (m.min-1)

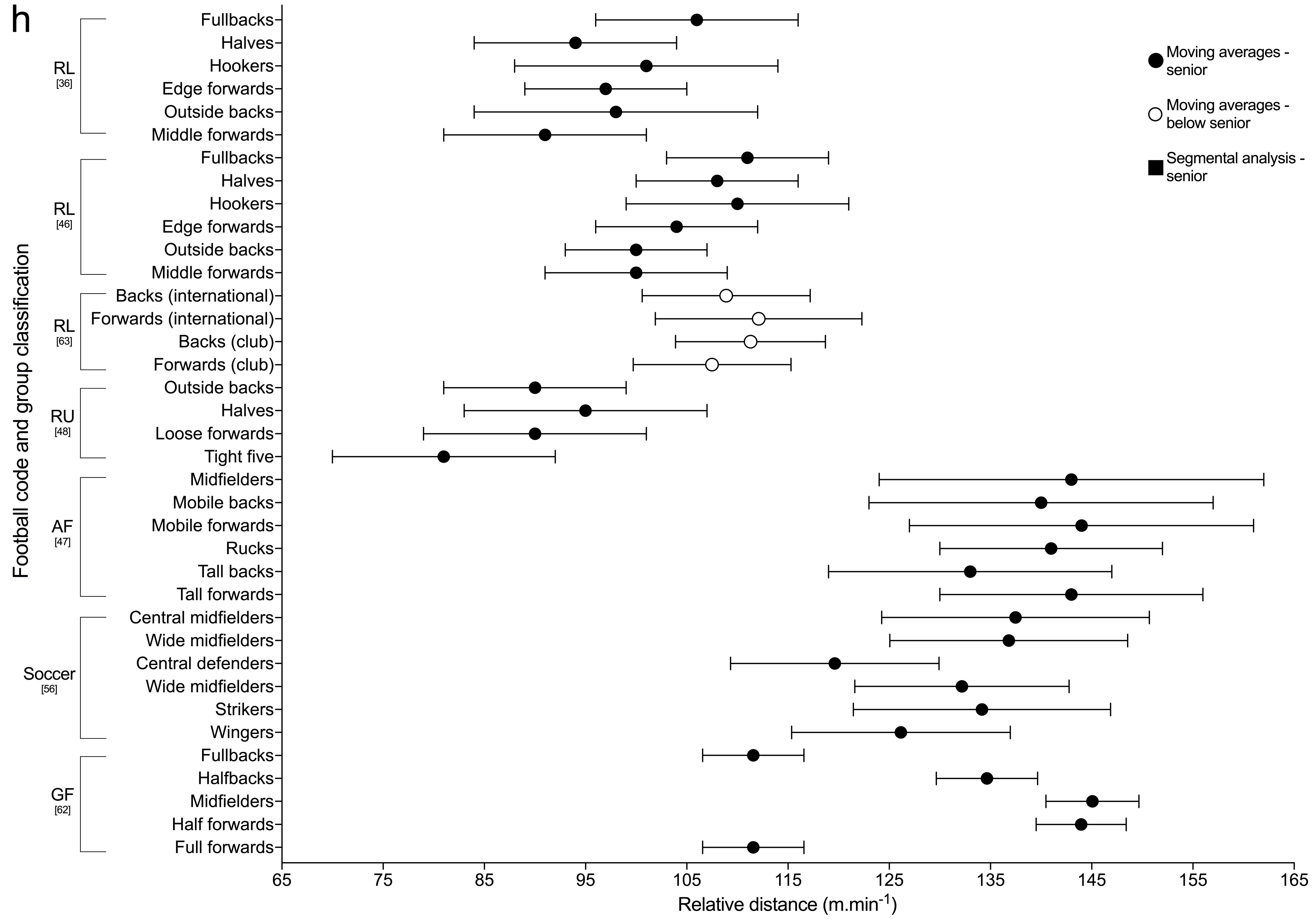
h
Football code and group classification
RL [36]
Fullbacks
Halves
Hookers
Edge forwards
Outside backs
Middle forwards
RL [46]
Fullbacks
Halves
Hookers
Edge forwards
Outside backs
Middle forwards
RL [63]
Backs (international)
Forwards (international)
Backs (club)
Forwards (club)
RU [48]
Outside backs
Halves
Loose forwards
Tight five
AF [47]
Midfielders
Mobile backs
Mobile forwards
Rucks
Tall backs
Tall forwards
Soccer [56]
Central midfielders
Wide midfielders
Central defenders
Wide midfielders
Strikers
Wingers
GF [62]
Fullbacks
Halfbacks
Midfielders
Half forwards
Full forwards
Moving averages - senior
Moving averages - below senior
Segmental analysis - senior
65
75
85
95
105
115
125
135
145
155
165
Relative distance (m.min-1)
