# Supplementary material for: The Use of Microtechnology to Quantify the Peak Match Demands of the Football Codes: A Systematic Review
Source: Sports Med. 2018 Aug 7;48(11):2549–75. doi: 10.1007/s40279-018-0965-6 (PMC6182461; doi:10.1007/s40279-018-0965-6)
Supplement: Supplementary file 2 — Figure S2. Duration specific peak average absolute acceleration/deceleration (AveAcc; m·s-2) in the football codes. a = 2-minutes, b = 3-minutes, c = 4-minutes, d = 6-minutes, e = 7-minutes, f = 8-minutes, g = 9-minutes. Data expressed as mean ± SD. RL = rugby league, RU = rugby union, AF = Australian Football [file 40279_2018_965_MOESM2_ESM.pdf]

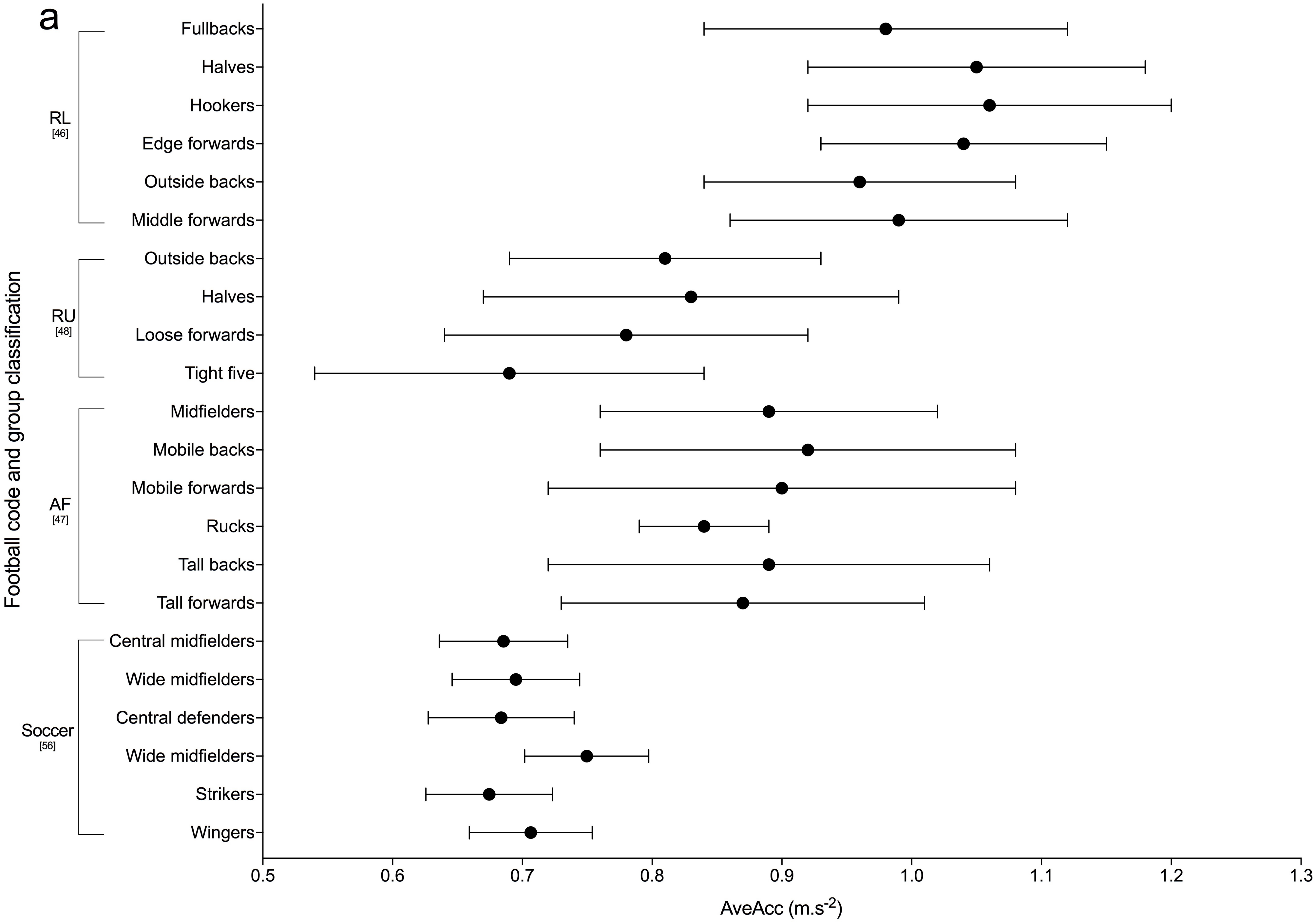
a
RL
[46]
Fullbacks
Halves
Hookers
Edge forwards
Outside backs
Middle forwards
RU
[48]
Outside backs
Halves
Loose forwards
Tight five
AF
[47]
Midfielders
Mobile backs
Mobile forwards
Rucks
Tall backs
Tall forwards
Soccer
[56]
Central midfielders
Wide midfielders
Central defenders
Wide midfielders
Strikers
Wingers
Football code and group classification
0.5
0.6
0.7
0.8
0.9
1.0
1.1
1.2
1.3
AveAcc (m.s-2)

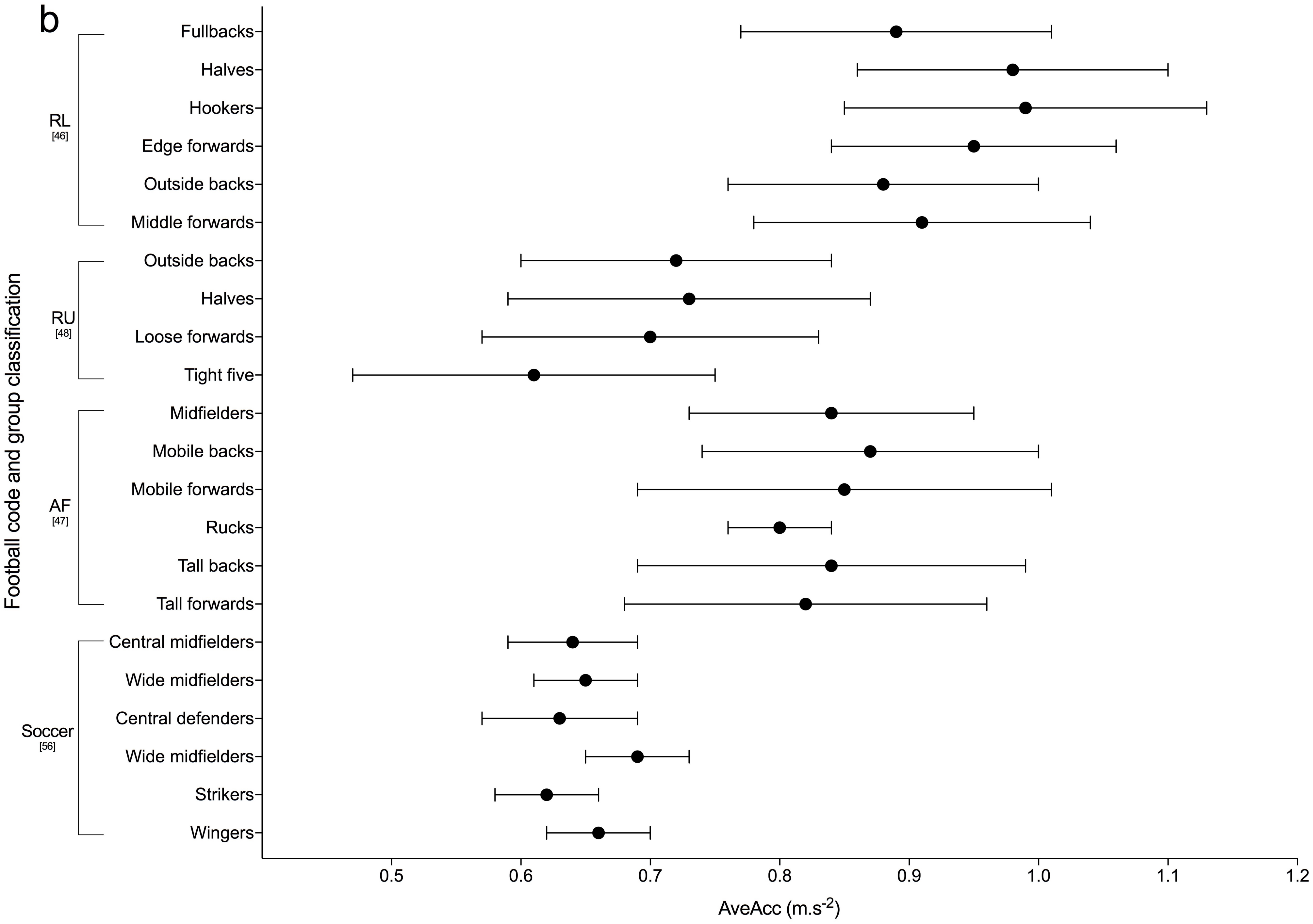
b
Football code and group classification
RL
[46]
Fullbacks
Halves
Hookers
Edge forwards
Outside backs
Middle forwards
RU
[48]
Outside backs
Halves
Loose forwards
Tight five
AF
[47]
Midfielders
Mobile backs
Mobile forwards
Rucks
Tall backs
Tall forwards
Soccer
[56]
Central midfielders
Wide midfielders
Central defenders
Wide midfielders
Strikers
Wingers
0.5
0.6
0.7
0.8
0.9
1.0
1.1
1.2
AveAcc (m.s-2)

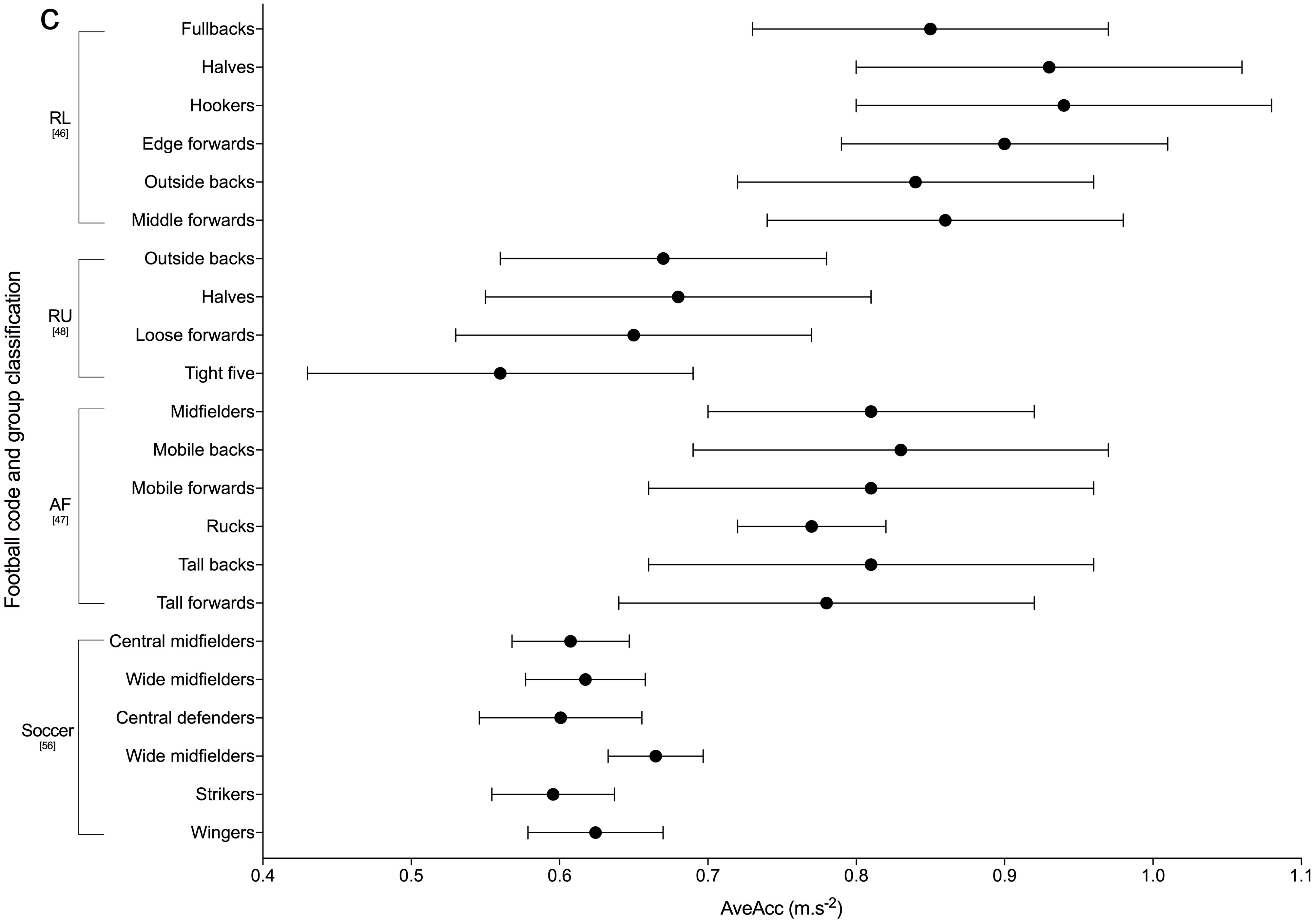

C
RL
[46]
Fullbacks
Halves
Hookers
Edge forwards
Outside backs
Middle forwards
RU
[48]
Outside backs
Halves
Loose forwards
Tight five
AF
[47]
Midfielders
Mobile backs
Mobile forwards
Rucks
Tall backs
Tall forwards
Soccer
[56]
Central midfielders
Wide midfielders
Central defenders
Wide midfielders
Strikers
Wingers
Football code and group classification
0.4
0.5
0.6
0.7
0.8
0.9
1.0
1.1
AveAcc (m.s-2)

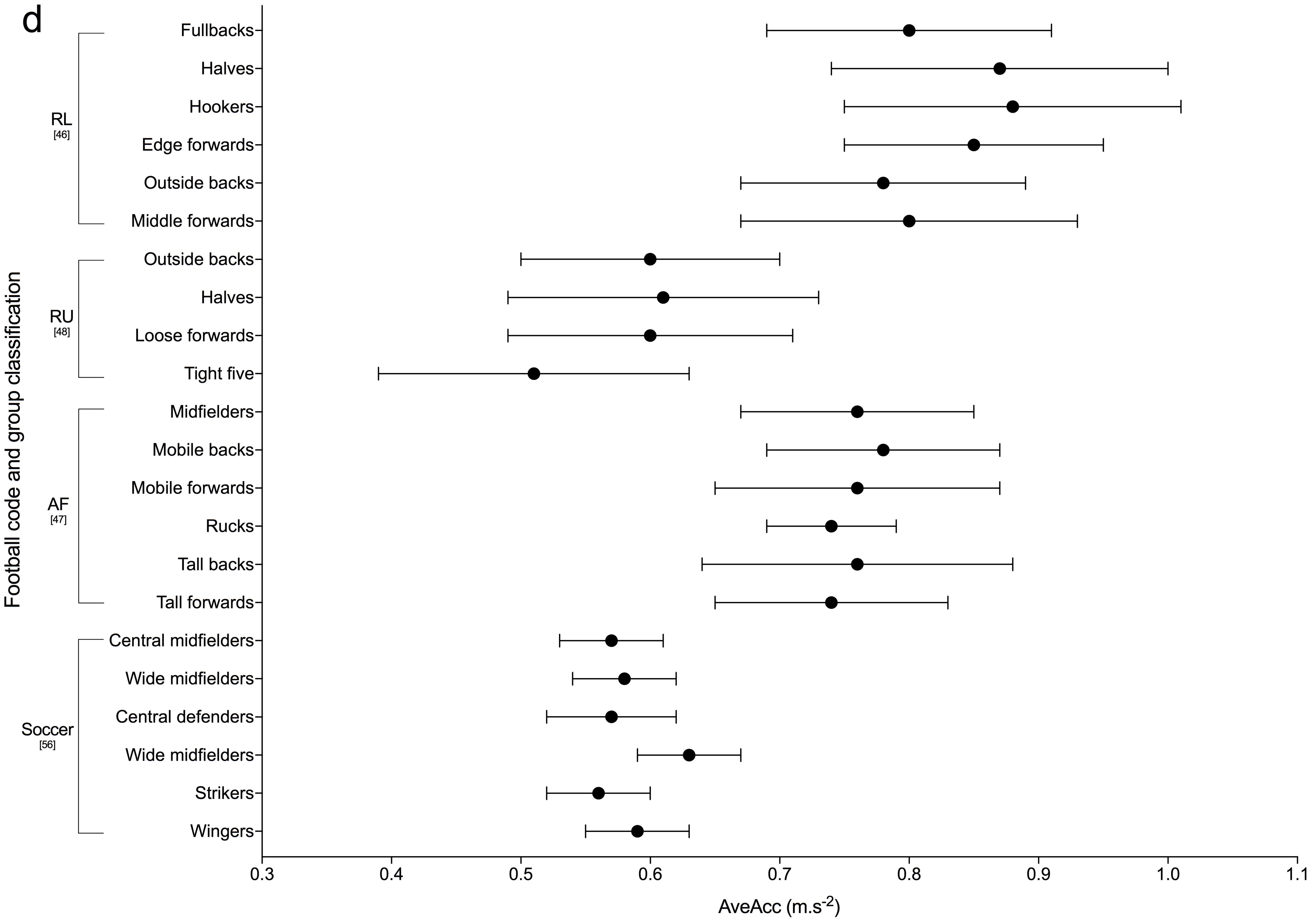
d
Football code and group classification
RL
[46]
Fullbacks
Halves
Hookers
Edge forwards
Outside backs
Middle forwards
RU
[48]
Outside backs
Halves
Loose forwards
Tight five
AF
[47]
Midfielders
Mobile backs
Mobile forwards
Rucks
Tall backs
Tall forwards
Soccer
[56]
Central midfielders
Wide midfielders
Central defenders
Wide midfielders
Strikers
Wingers
0.3
0.4
0.5
0.6
0.7
0.8
0.9
1.0
1.1
AveAcc (m.s-2)

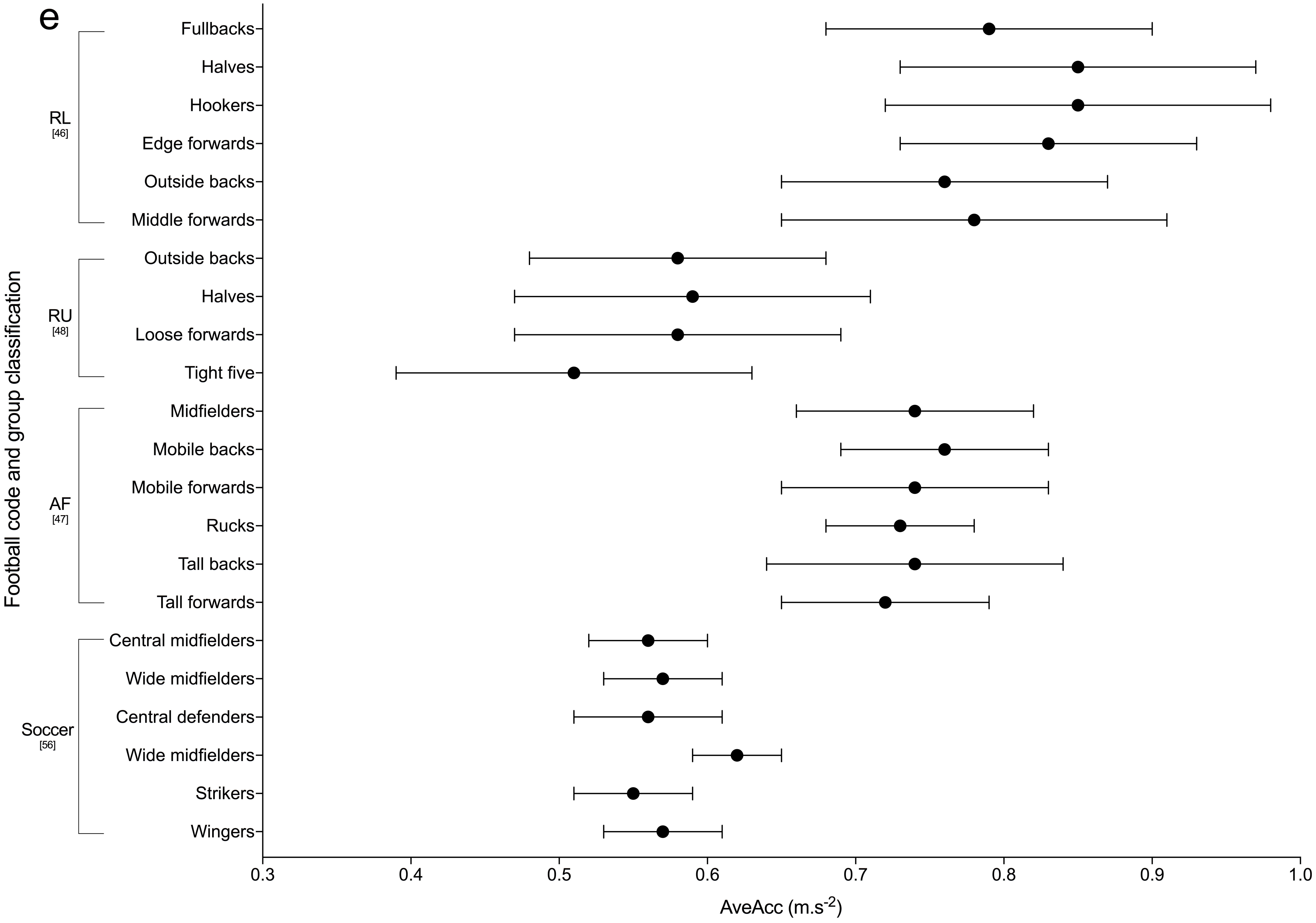
e
RL
[46]
Fullbacks
Halves
Hookers
Edge forwards
Outside backs
Middle forwards
RU
[48]
Outside backs
Halves
Loose forwards
Tight five
AF
[47]
Midfielders
Mobile backs
Mobile forwards
Rucks
Tall backs
Tall forwards
Soccer
[56]
Central midfielders
Wide midfielders
Central defenders
Wide midfielders
Strikers
Wingers
Football code and group classification
0.3
0.4
0.5
0.6
0.7
0.8
0.9
1.0
AveAcc (m.s-2)

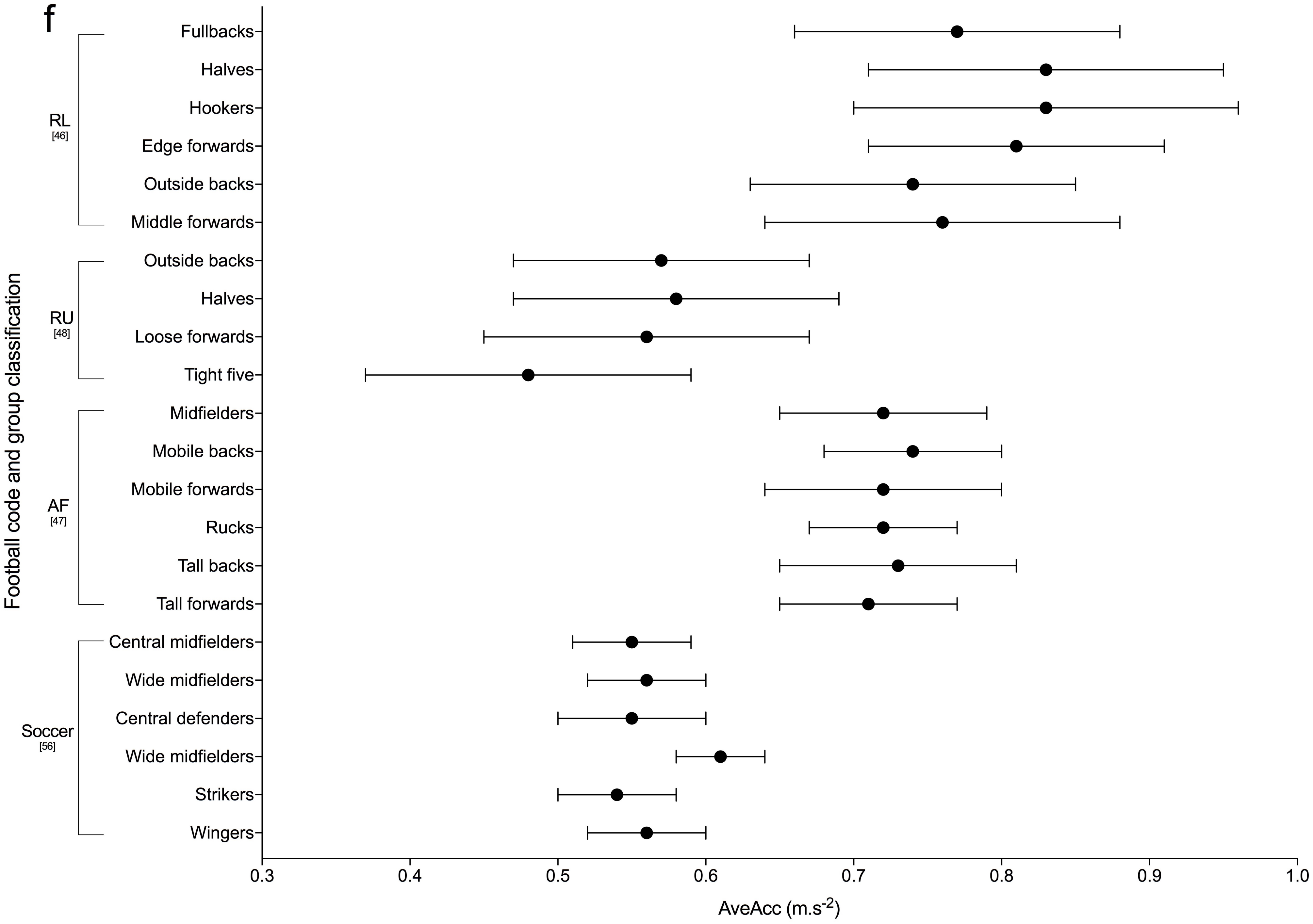
f
RL
[46]
Fullbacks
Halves
Hookers
Edge forwards
Outside backs
Middle forwards
RU
[48]
Outside backs
Halves
Loose forwards
Tight five
AF
[47]
Midfielders
Mobile backs
Mobile forwards
Rucks
Tall backs
Tall forwards
Soccer
[56]
Central midfielders
Wide midfielders
Central defenders
Wide midfielders
Strikers
Wingers
Football code and group classification
0.3
0.4
0.5
0.6
0.7
0.8
0.9
1.0
AveAcc (m.s-2)

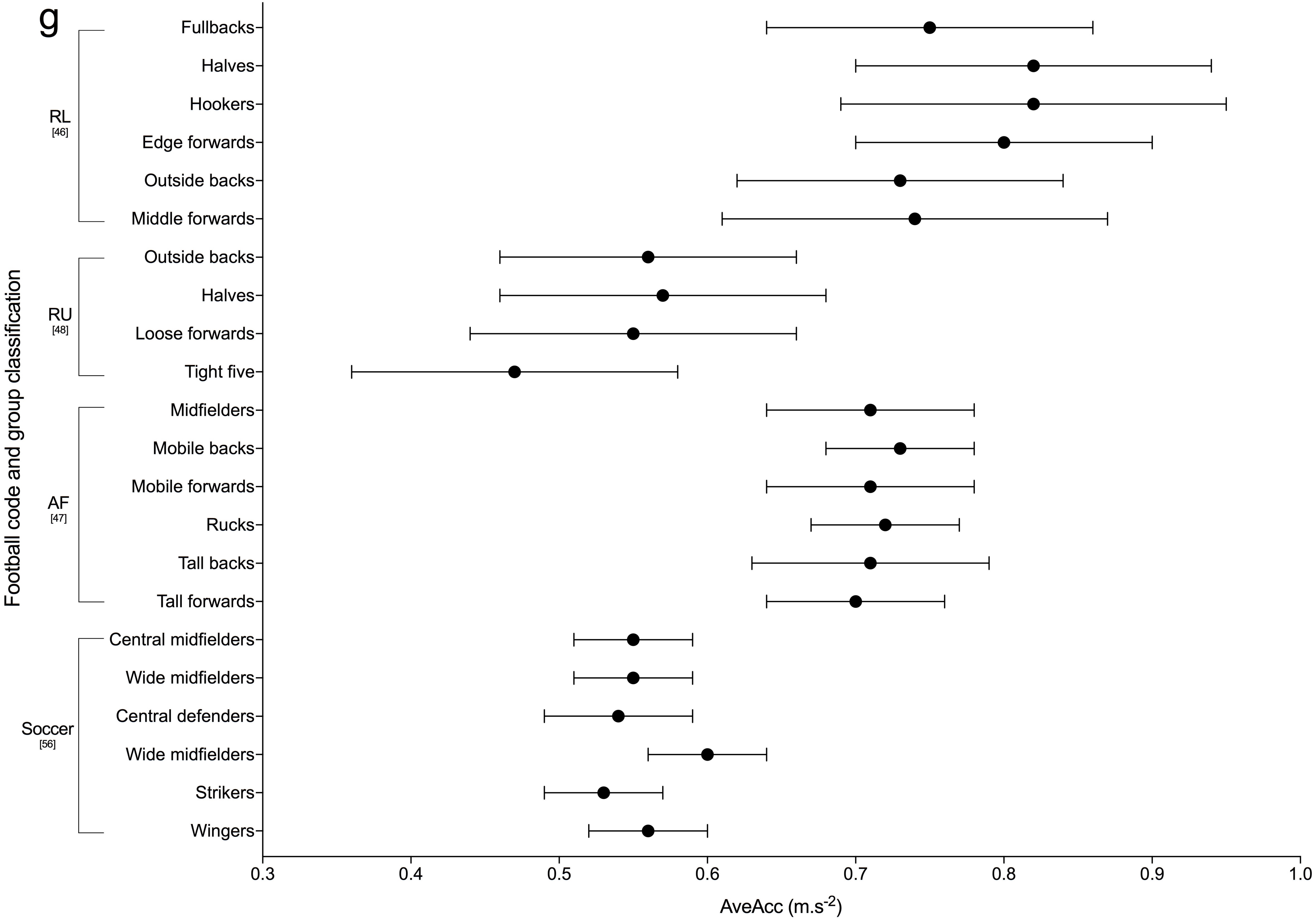
g
Fullbacks
Halves
Hookers
Edge forwards
Outside backs
Middle forwards
RL
[46]
Outside backs
Halves
Loose forwards
Tight five
RU
[48]
Midfielders
Mobile backs
Mobile forwards
Rucks
Tall backs
Tall forwards
AF
[47]
Central midfielders
Wide midfielders
Central defenders
Wide midfielders
Strikers
Wingers
Soccer
[56]
Football code and group classification
0.3
0.4
0.5
0.6
0.7
0.8
0.9
1.0
AveAcc (m.s-2)
