# Supplementary material for: The Use of Microtechnology to Quantify the Peak Match Demands of the Football Codes: A Systematic Review
Source: Sports Med. 2018 Aug 7;48(11):2549–75. doi: 10.1007/s40279-018-0965-6 (PMC6182461; doi:10.1007/s40279-018-0965-6)
Supplement: Supplementary file 3 — Figure S3. Duration-specific peak metabolic power (W·kg-1) in the football codes. a = 2-minutes, b = 3-minutes, c = 4-minutes, d = 6-minutes, e = 7-minutes, f = 8-minutes, g = 9-minutes. Data expressed as mean ± SD. RL = rugby league, RU = rugby union, AF = Australian Football, R7 = rugby sevens [file 40279_2018_965_MOESM3_ESM.pdf]

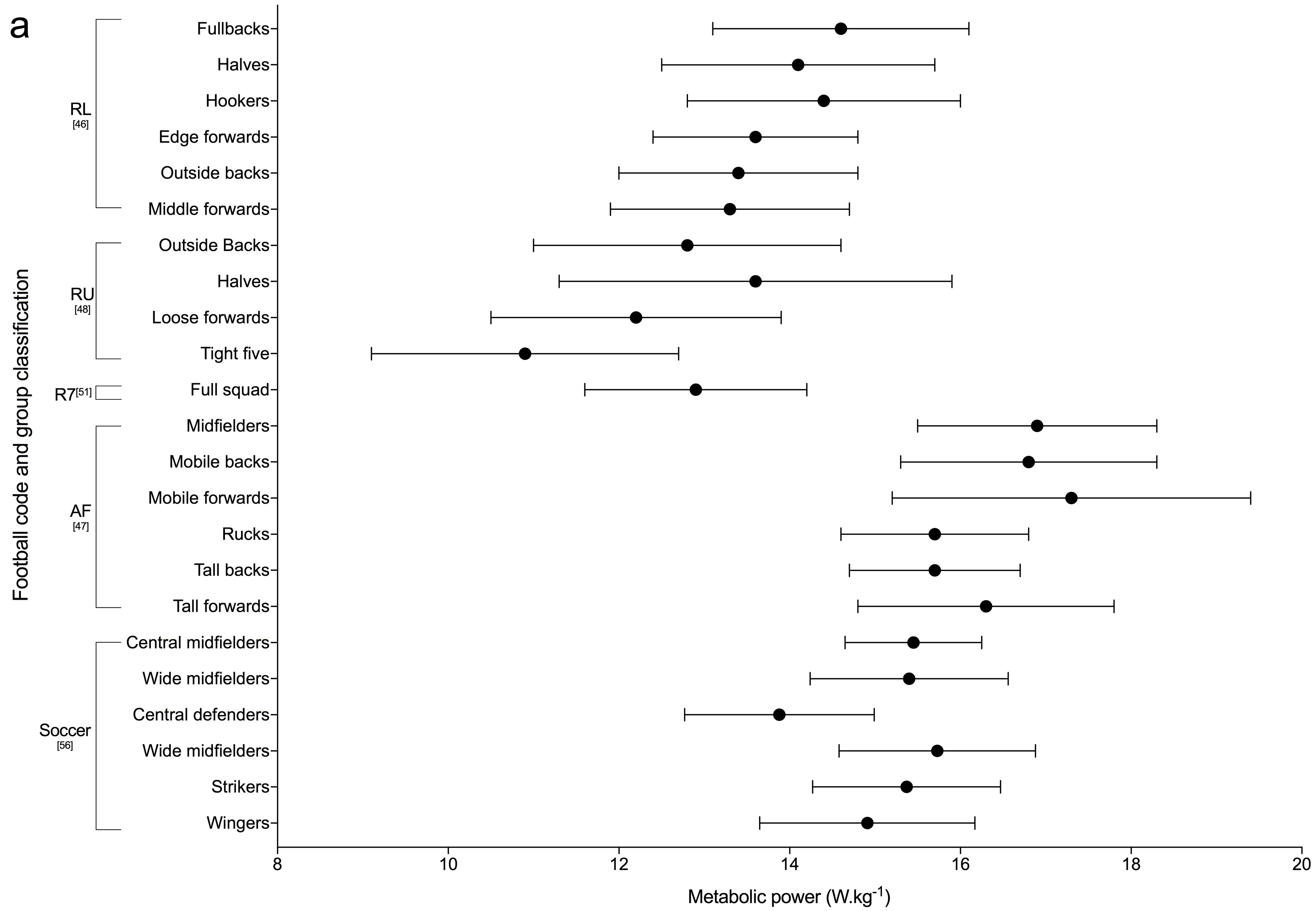

a
RL [46]
Fullbacks
Halves
Hookers
Edge forwards
Outside backs
Middle forwards
RU [48]
Outside Backs
Halves
Loose forwards
Tight five
R7[51]
Full squad
AF [47]
Midfielders
Mobile backs
Mobile forwards
Rucks
Tall backs
Tall forwards
Soccer [56]
Central midfielders
Wide midfielders
Central defenders
Wide midfielders
Strikers
Wingers
Football code and group classification
8
10
12
14
16
18
20
Metabolic power (W.kg-1)

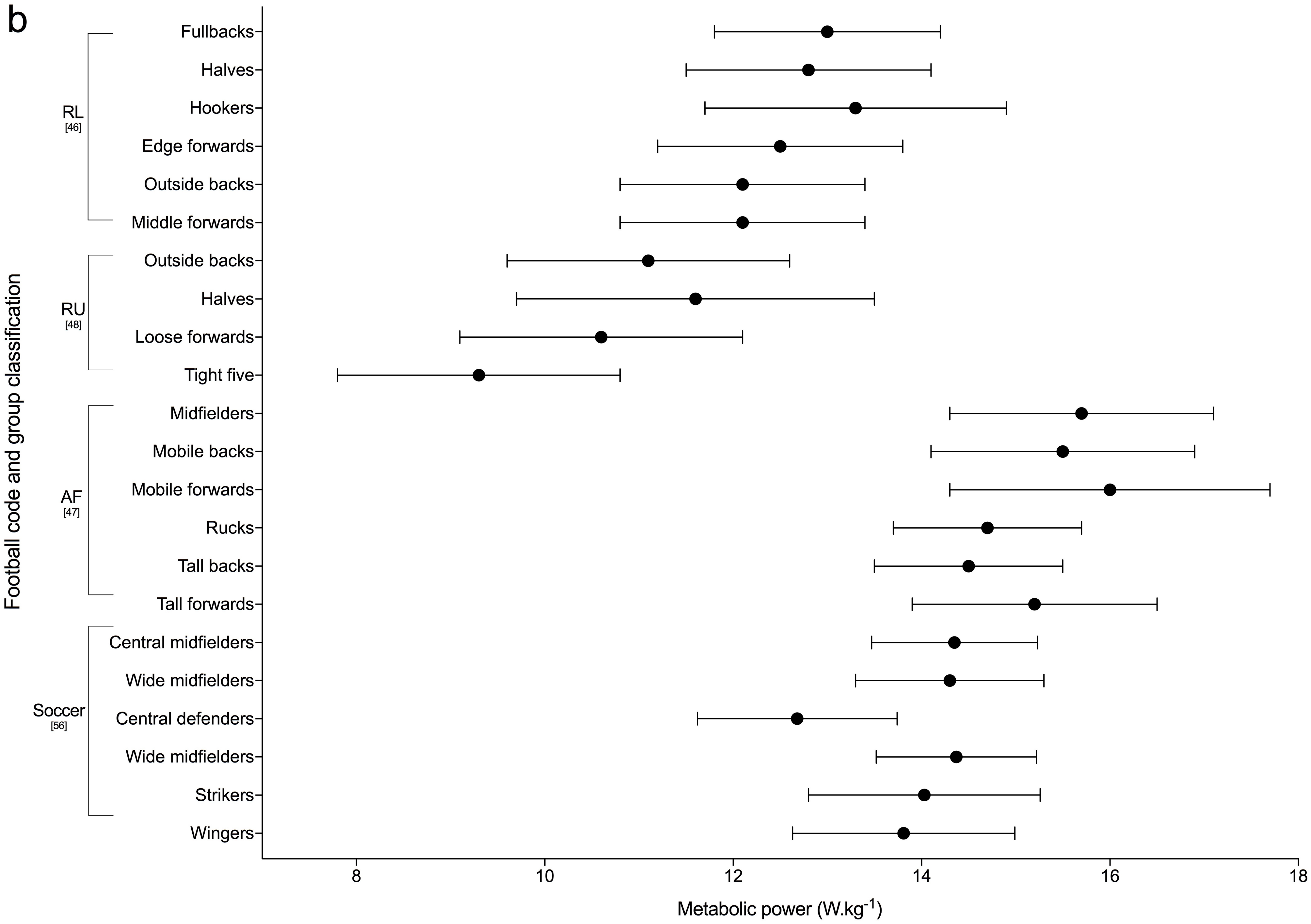
b
RL
[46]
Fullbacks
Halves
Hookers
Edge forwards
Outside backs
Middle forwards
RU
[48]
Outside backs
Halves
Loose forwards
Tight five
AF
[47]
Midfielders
Mobile backs
Mobile forwards
Rucks
Tall backs
Tall forwards
Soccer
[56]
Central midfielders
Wide midfielders
Central defenders
Wide midfielders
Strikers
Wingers
Football code and group classification
8
10
12
14
16
18
Metabolic power (W.kg-1)

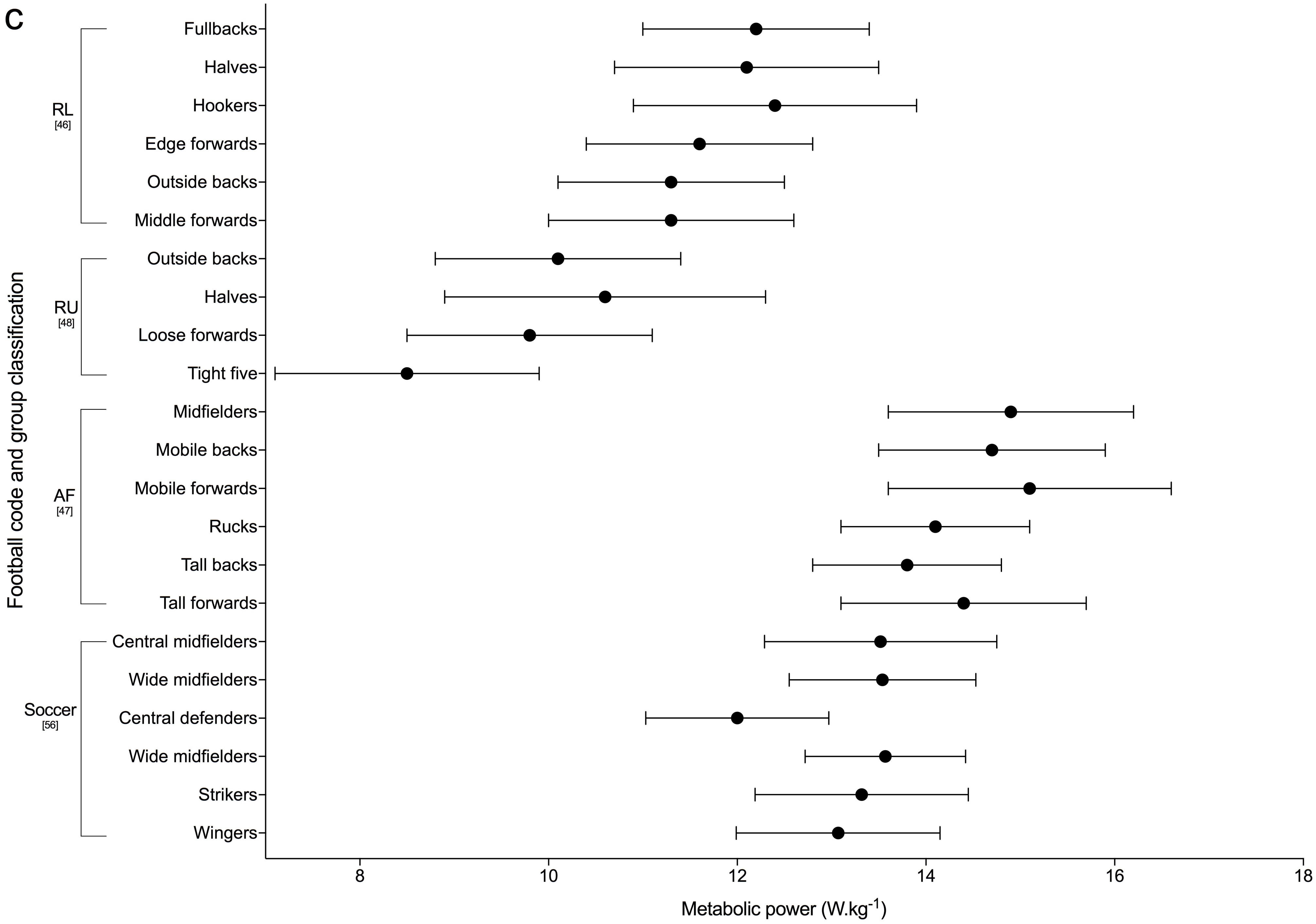
C
RL
[46]
Fullbacks
Halves
Hookers
Edge forwards
Outside backs
Middle forwards
RU
[48]
Outside backs
Halves
Loose forwards
Tight five
AF
[47]
Midfielders
Mobile backs
Mobile forwards
Rucks
Tall backs
Tall forwards
Soccer
[56]
Central midfielders
Wide midfielders
Central defenders
Wide midfielders
Strikers
Wingers
Football code and group classification
8
10
12
14
16
18
Metabolic power (W.kg-1)

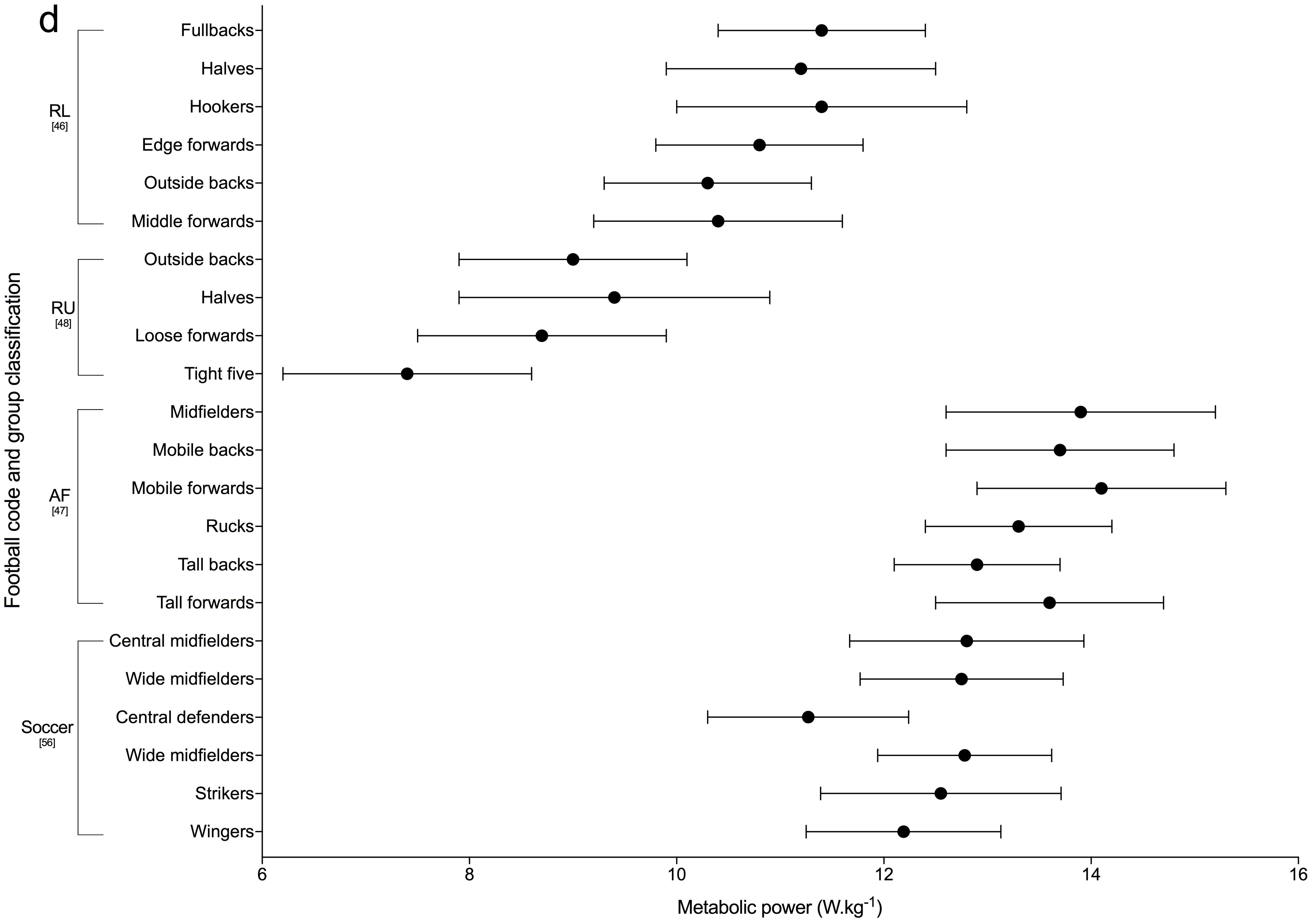

d
RL
[46]
Fullbacks
Halves
Hookers
Edge forwards
Outside backs
Middle forwards
RU
[48]
Outside backs
Halves
Loose forwards
Tight five
AF
[47]
Midfielders
Mobile backs
Mobile forwards
Rucks
Tall backs
Tall forwards
Soccer
[56]
Central midfielders
Wide midfielders
Central defenders
Wide midfielders
Strikers
Wingers
Football code and group classification
6
8
10
12
14
16
Metabolic power (W.kg-1)

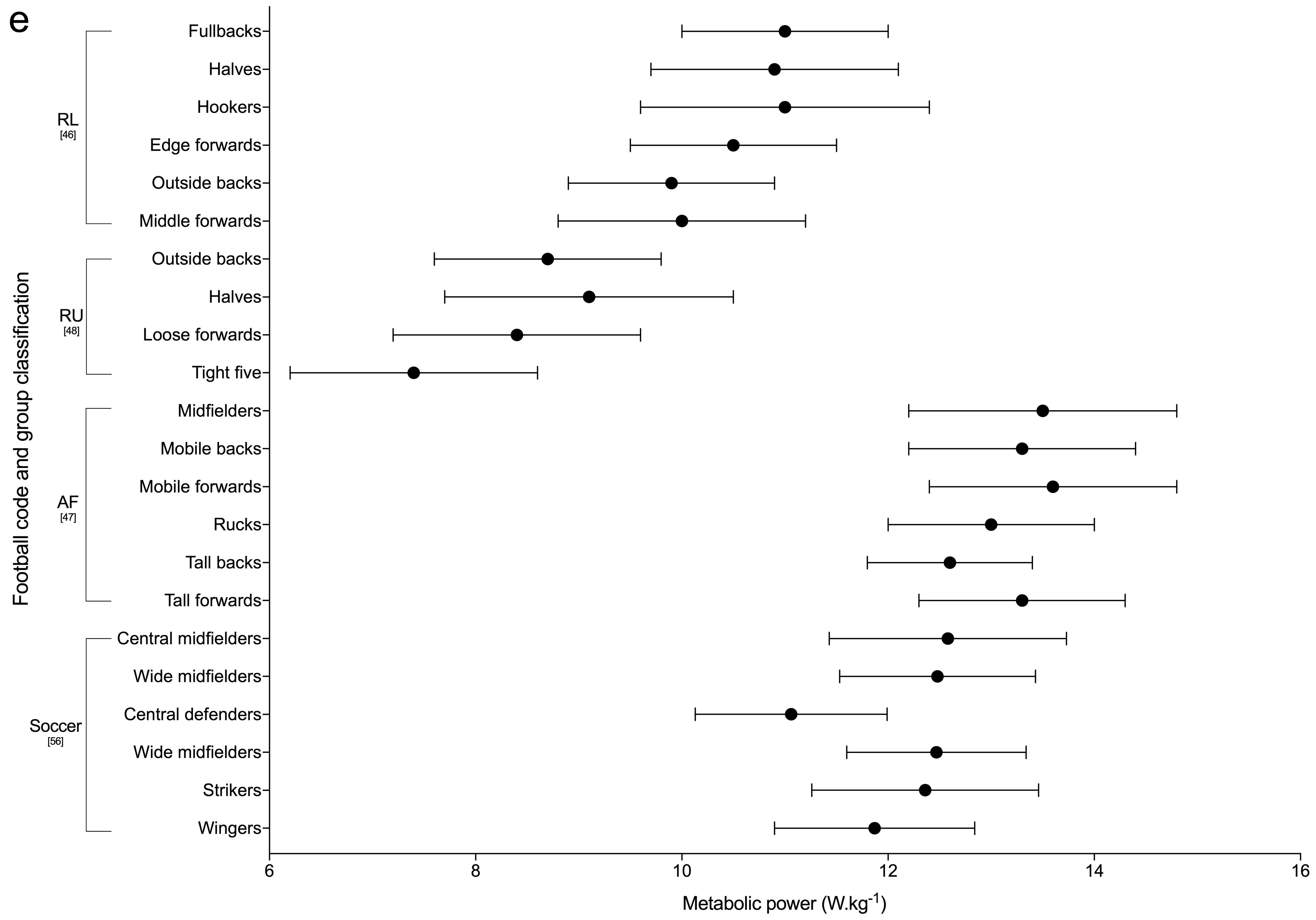

e
Fullbacks
Halves
Hookers
Edge forwards
Outside backs
Middle forwards
Outside backs
Halves
Loose forwards
Tight five
Midfielders
Mobile backs
Mobile forwards
Rucks
Tall backs
Tall forwards
Central midfielders
Wide midfielders
Central defenders
Wide midfielders
Strikers
Wingers
RL [46]
RU [48]
AF [47]
Soccer [56]
Football code and group classification
6
8
10
12
14
16
Metabolic power (W.kg-1)

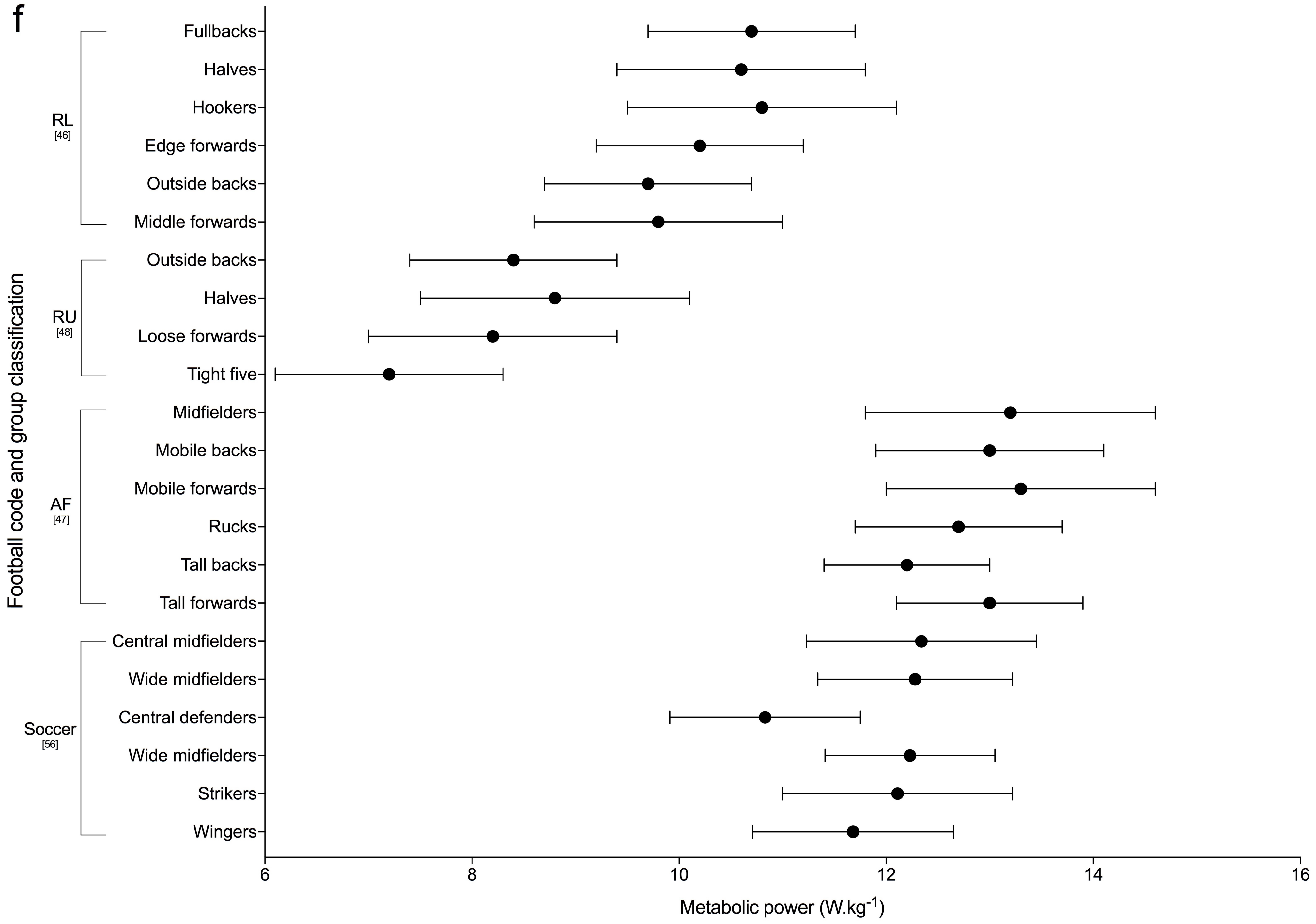

f
Football code and group classification
RL
[46]
Fullbacks
Halves
Hookers
Edge forwards
Outside backs
Middle forwards
RU
[48]
Outside backs
Halves
Loose forwards
Tight five
AF
[47]
Midfielders
Mobile backs
Mobile forwards
Rucks
Tall backs
Tall forwards
Soccer
[56]
Central midfielders
Wide midfielders
Central defenders
Wide midfielders
Strikers
Wingers
6
8
10
12
14
16
Metabolic power (W.kg-1)

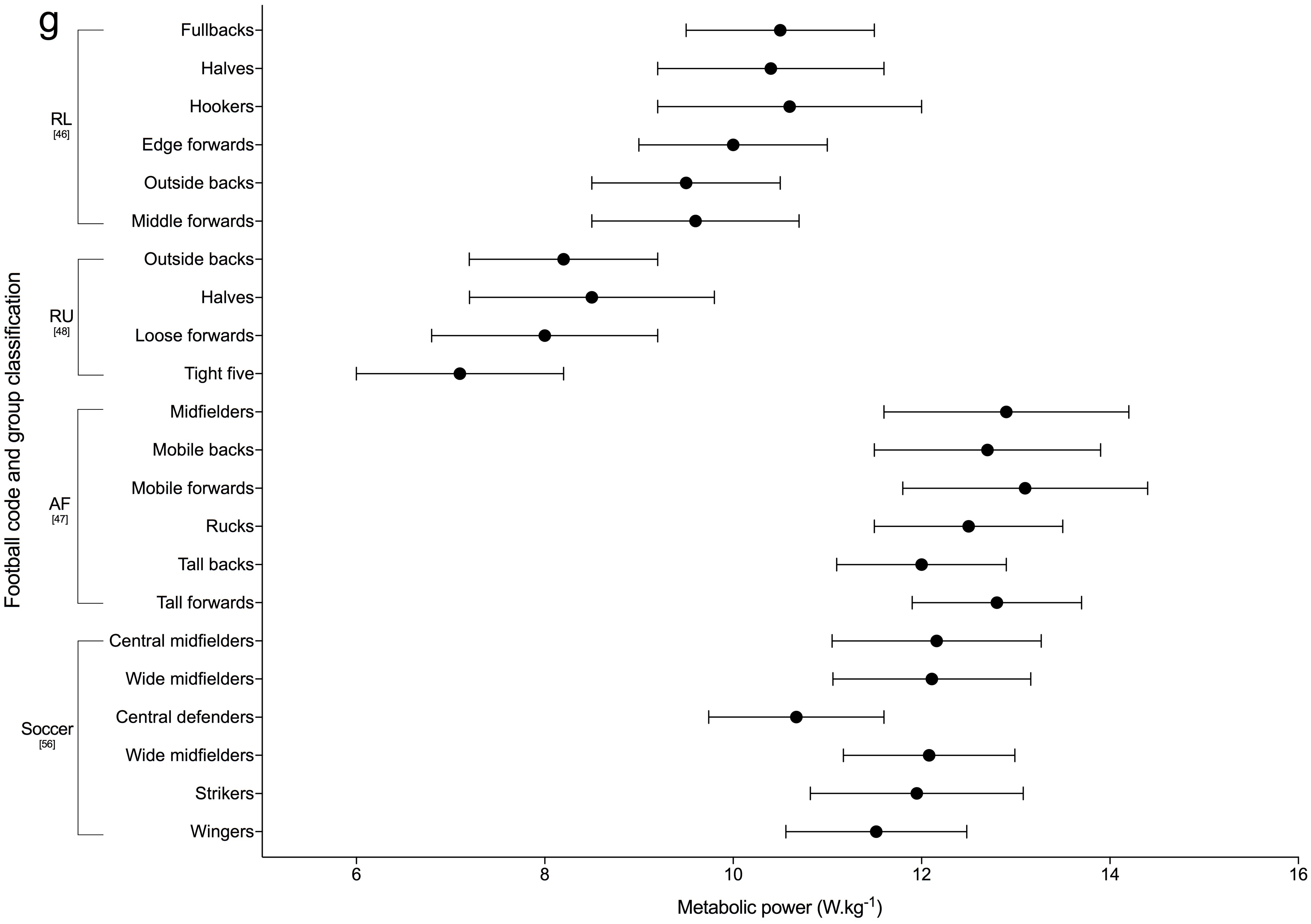

g
RL
[46]
Fullbacks
Halves
Hookers
Edge forwards
Outside backs
Middle forwards
RU
[48]
Outside backs
Halves
Loose forwards
Tight five
AF
[47]
Midfielders
Mobile backs
Mobile forwards
Rucks
Tall backs
Tall forwards
Soccer
[56]
Central midfielders
Wide midfielders
Central defenders
Wide midfielders
Strikers
Wingers
Football code and group classification
6
8
10
12
14
16
Metabolic power (W.kg-1)
